# Long-Circulating Liposomes Codelivering Amphotericin B and Retinoic Acid for Cutaneous Leishmaniasis Treatment

**DOI:** 10.1021/acsomega.5c06156

**Published:** 2025-10-07

**Authors:** Thais T. Santos, Eduardo B. Lages, Tiago N. Q. Ricotta, Leandro G. de Oliveira, Guilherme S. Ramos, Julie Burlot, Sonia Abreu, Pierre Chaminade, François-Xavier Legrand, Pauline Tran, Claudine Deloménie, Virgínia M. R. Vallejos, Doumet Georges Helou, Raquel M. de Almeida, Celso M. Queiroz-Junior, Gabriel B. M. Teobaldo, Cristiano L. P. de Oliveira, Lucas A. M. Ferreira, Marta M. G. Aguiar, Sébastien Pomel, Frédéric Frézard

**Affiliations:** † Institute of Biological Sciences, 28114 Federal University of Minas Gerais (UFMG), 31270-901 Belo Horizonte, Brazil; ‡ Université Paris-Saclay, CNRS BioCIS, 91400 Orsay, France; § Faculty of Pharmacy, Federal University of Minas Gerais (UFMG), 31270-901 Belo Horizonte, Brazil; ∥ Université Paris-Saclay, UMS-IPSIT Animex, INSERM, CNRS, Ingénierie et Plateformes au Service de l’Innovation Thérapeutique, 91400 Orsay, France; ⊥ Lipides: Systèmes Analytiques et Biologiques, Université Paris-Saclay, 91400 Orsay, France; # Université Paris-Saclay, CNRS, Institut Galien Paris-Saclay, 91400 Orsay, France; ∇ Université Paris-Saclay, UMS-IPSIT ACTAGen, INSERM, CNRS, Ingénierie et Plateformes au Service de l’Innovation Thérapeutique, 91400 Orsay, France; ○ Université Paris Cité, INSERM UMR1149, Centre de Recherche sur l’Inflammation (CRI), Paris 75018, France; ◆ Instituto de Física, 28133 Universidade de São Paulo, 05508-0900 São Paulo, Brazil

## Abstract

Leishmaniasis is a neglected tropical disease that mainly affects socially vulnerable populations. It is caused by various *Leishmania* species, and manifests primarily as cutaneous (CL) or visceral leishmaniasis (VL). AmBisome, a commercial formulation of amphotericin B (AmB) with conventional liposomes, is the most effective for VL treatment, but its efficacy is limited in some CL cases and immunocompromised patients. This work evaluated a new PEGylated liposomal formulation (LAmB-RA) coencapsulating AmB and retinoic acid (RA), as an immunomodulator, for CL treatment. LAmB-RA displayed a mean size of 125 nm, a polydispersity index <0.2, and high encapsulation efficiencies for AmB (97.1%) and RA (94.7%). Although SAXS analysis indicates that RA did not induce major structural rearrangements in the liposomal bilayer, comparison of circular dichroism spectra between LAmB-RA and the liposomal AmB formulation without RA (LAmB) revealed slightly different aggregated states of AmB. In addition, LAmB-RA showed a significantly reduced hemolytic activity, compared to LAmB. Pharmacokinetic analysis of the PEGylated formulations showed higher *C*_max_ and prolonged plasma exposure of AmB compared to AmBisome. In Leishmania *major*-infected mice, LAmB-RA significantly reduced lesion size and parasite burden (99%) versus untreated control. In the *Leishmania amazonensis* model, it markedly inhibited lesion progression and was the only treatment to significantly reduce parasite load in the spleen (by 60%). Additionally, LAmB-RA promoted an increase in the IFN-γ/IL-10 ratio in antigen-stimulated splenocytes compared to LAmB, indicating a Th1-skewed immune response. These results support LAmB-RA as a promising therapeutic strategy for CL, combining prolonged circulation and enhanced efficacy.

## Graphical abstract

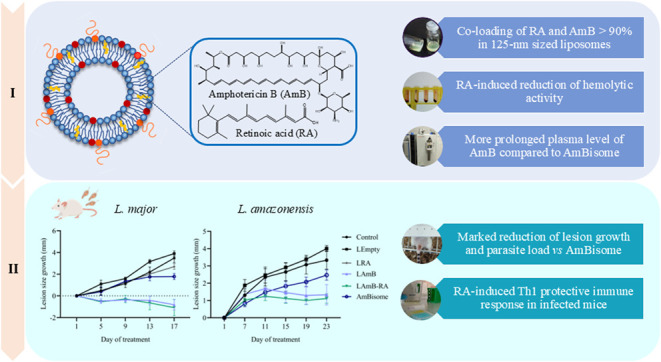

## 1 Introduction

Leishmaniasis represents a group of neglected tropical and subtropical diseases affecting socially vulnerable and unassisted populations. Different clinical manifestations depending on the *Leishmania* species and the host’s immune status can be observed, with the most common clinical forms being cutaneous leishmaniasis (CL), usually manifested as single-skin lesions, mucocutaneous leishmaniasis (MCL) when mucous membranes are affected and visceral leishmaniasis (VL), the most severe form which is lethal in absence of treatment. This latter form occurs when the parasites are found in the liver, spleen, and bone marrow.^1,2^ The World Health Organization (WHO) reported 205,986 new CL cases and 12,842 new VL cases in 2022,^3^ significantly impacting public health.

These diseases are caused by more than 20 species of *Leishmania* protozoa belonging to the *Trypanosomatidae* family.^2^ Their transmission to mammals, including humans, occurs during the blood meal of infected female phlebotomine sandflies. Once introduced into the host’s dermis, *Leishmania* is phagocytosed by Mononuclear Phagocytic System (MPS) cells, i.e., neutrophils, monocytes, dendritic cells, and the macrophages, which are predominant *Leishmania* host cells.^4^ After phagocytosis, *Leishmania* proliferates in a parasitophorous vacuole which derives from the phagosome fusion with lysosomes, leading to cell rupture, new MPS cells infection, and disease progression.^4,5^

While MPS cells can usually eliminate infectious agents, *Leishmania* parasites are able to circumvent the host cell’s defense. In this scenario, a competent immune system is essential to control the infection. It has been proposed that a predominant cellular immune response with T helper 1 (Th1) characteristics, involving activation of CD4^+^ and CD8^+^ T lymphocytes and secretion of cytokines such as interleukin (IL)-12, interferon-γ (IFN-γ) and tumor necrosis factor-α (TNF-α), are necessary for a resolving response. This pro-inflammatory response activates macrophages, producing reactive oxygen and nitrogen species for parasite elimination.^5^ On the other hand, T helper 2 (Th2) responses, characterized by the production of anti-inflammatory cytokines such as IL-4 and IL-10, are related to disease progression. However, there is a delicate balance between Th1/Th2 responses. Although the predominance of Th1-type responses is related to leishmaniasis control, its exacerbation has been associated with more severe symptoms. Therefore, introducing immunomodulators in leishmaniasis treatment represents an interesting approach to influence this balance.^6^

For more than 70 years, pentavalent antimonials have been the mainstay for leishmaniasis treatment despite their high cardiac, hepatic, and renal toxicity, as well as increasing resistance reports.^7^ Patients can also be treated with amphotericin B (AmB) (in micellar or liposomal form), paromomycin, or miltefosine.^5,8,9^ It should be noted that these drugs have toxicity, cost, and resistance limitation issues.^1,10^

AmB is the most potent antileishmanial currently available. The main mechanism of action reported in the literature for AmB involves the formation of pores in membranes due to its interaction to ergosterol, a sterol specifically present in *Leishmania* plasma membrane.^11,12^ Despite being a highly effective drug in leishmaniasis treatment, AmB has limitations related to toxicity, especially renal toxicity, due to some interaction with cholesterol in mammalian cells. In an attempt to reduce nephrotoxicity, lipid formulations have been developed.^12^ In this way, AmBisome, a formulation of AmB with conventional liposomes, is considered as the safest and the most effective drug against VL; however, its efficacy is limited in some CL cases and leishmaniasis-HIV (human immunodeficiency virus) coinfection.^13,14^ Moreover, some rare cases of AmB-resistant species for CL^15^ and VL^16,17^ have been reported. For this reason, there has been an intense search in recent years of new delivery strategies of conventional or novel chemotherapeutics, using for instance electrospun fibers, hydrogels, and other polymeric scaffolds, such as microneedle patches. Recent approaches also highlight the potential of cell therapy with mesenchymal stromal cells, due to their bidirectional interaction with the immune system and pathogens.^18^

Among delivery systems, nanotechnology-based formulations remain one of the most explored alternatives for CL treatment. One strategy in this field has consisted in modifying AmB liposomes surface with 1,2-distearoyl-*sn*-glycero-3-phosphoethanolamine-*N*-[methoxy (polyethylene glycol)-2000] (DSPE-PEG_2000_), allowing greater efficacy in a CL murine model after parenteral and oral administrations.^19^ Another recent strategy is the improvement of conventional chemotherapy through association with an immunomodulator.^20,21^ In this context, retinoic acid (RA), a micronutrient obtained from retinol (vitamin A), seems promising. Recent studies evaluating vitamin D_3_/RA and chenodeoxycholic acid/RA combinations showed downregulation of the tryptophan–aspartate-containing coat (TACO) gene and parasite load reduction *in vitro* and *in vivo*.^10,22^ A marked Th2 immune response inhibition was also detected, with an increase in the Th1 response.^10,22–24^ Moreover, vitamin A deficiency has been reported in patients with leishmaniasis, suggesting an impact on the disease’s clinical course.^25^

In addition to an immunomodulatory effect, RA exhibits direct antileishmanial activity, as demonstrated in recent *in vitro* studies.^26–29^ RA supplementation in *Leishmania donovani*-infected macrophages restored the cellular cholesterol content and reduced the parasite load. Mechanistically, this effect is mediated by increased mRNA expression of Niemann-Pick type C1 (NPC1) and C2 (NPC2) genes, which are responsible for lysosomal cholesterol trafficking.^26,27,29^ Concurrently, RA acts directly on the parasite by targeting the ergosterol biosynthesis pathway. Prakash et al.^28^ demonstrated, through molecular docking and *in vitro* studies, that RA inhibits sterol 24-C-methyltransferase (SMT), a key enzyme responsible for converting zymosterol into ergosterol, a vital component of the *Leishmania* membrane. Given these findings, authors suggested a dual function for RA: immunomodulatory and direct parasiticidal actions. RA has also been investigated for its potential to promote healing and stimulate fibroblast proliferation, with reduced skin reactions and irritation through encapsulation in nanosystems.^30^

Conversely, data pointing to an anti-inflammatory effect (Th2) have also been reported in the literature, with RA,^31,32^ but also with vitamin A^33,34^ treatment. Thus, because of the conflicting reports on the immunomodulary action of RA, one cannot anticipate whether the association of AmB with RA would promote a Th1 or Th2 immune response.

Even though RA has a therapeutic interest, retinoids in their natural form are unstable, have low bioavailability, and are highly lipophilic (log *P* = 6.3).^35^ To address these issues, nanocarriers can be used to improve RA physicochemical characteristics, protecting it from early degradation and favoring its delivery to target cells (MPS cells, particularly macrophages in the dermis). It is also worth highlighting that coincorporating different drugs within a single nanosystem can enhance treatment effectiveness through a synergistic effect, while also helping to delay the development of drug resistance.^20^

From this perspective, this paper introduces the innovative combination of these two drugs into a single formulation, leveraging their synergistic potential for a more effective CL therapy. To the best of our knowledge, this coencapsulation strategy is unprecedented in the literature, and no PEGylated liposomal product has been approved for this specific indication. This research aims to (i) develop and characterize the liposomal formulation coincorporating AmB and RA (LAmB-RA); (ii) investigate its hemolytic activity *in vitro*; (iii) perform a pharmacokinetic study of AmB; and (iv) assess its therapeutic efficacy and the resulting immune response in murine models of CL caused by *Leishmania major* and *Leishmania amazonensis*.

## 2 Materials and Methods

### 2.1 Materials

Cholesterol (CHOL) was purchased from Sigma-Aldrich (St. Louis, MO, USA). Hydrogenated soybean phosphatidylcholine (HSPC), distearoylphosphatidylglycerol (DSPG), and DSPE-PEG_2000_ were obtained from Lipoid (Ludwigshafen, Germany). AmBisome was provided by Gilead Science Inc. (Foster City, CA, USA), and Anforicin B by Laboratório Cristália (Itapira, São Paulo, Brazil). AmB (active pharmaceutical ingredient) was kindly provided by Laboratório Cristália (Itapira, São Paulo, Brazil). All-trans-retinoic acid (ATRA) was purchased from Santa Cruz Biotechnology (Dallas, TX, USA). All other chemicals and reagents used in this study were of analytical grade.

### 2.2 Development of LAmB-RA Formulation

LAmB-RA formulations were prepared through the incorporation of AmB and RA into preformed PEGylated liposomes made from HSPC:CHOL:DSPG:DSPE-PEG-_2000_ (5:2.5:2:0.5 molar ratio), using a modification of the method described by Ramos et al.^19^ Briefly, a lipid film was first prepared and then hydrated with deionized water to form multilamellar liposomes at a final lipid concentration of 50 mM. These liposomes were transformed into unilamellar vesicles through repeated extrusions (5 times) across 200 and 100 nm pore-size polycarbonate membranes (Lipex Extruder, Burnaby, BC, Canada). RA was first solubilized at 0.625 or 1.25 mg/mL with NaOH 0.1 M and protected from light for drug coincorporation. Immediately after solubilization, 0.8 mL from RA-solution was added to 10 mg AmB under magnetic stirring. After that, 2 mL of prewarmed (60 °C) liposome suspension was added to the AmB-RA solution. The drug/liposome mixture was incubated at 60 °C for 1 min 45 s, followed by pH adjustment to 6.8 by adding 0.1 M HCl/HEPES buffer solution. The final step was an incubation for 5 min at 60 °C. Liposomes encapsulating AmB (LAmB) or RA (LRA) separately were prepared using the same protocol, except for adding one of the drugs. Empty liposomes (LEmpty) were also prepared using this protocol, but in the absence of any drug.

### 2.3 Liposomes Physicochemical Characterization

#### 2.3.1 Mean Hydrodynamic Diameter, Polydispersity Index, Zeta Potential, and Morphology

Nanocarriers’ mean hydrodynamic diameter (size) and polydispersity index (PdI) were determined by dynamic light scattering (DLS), with a fixed angle of 90° and a temperature of 25 °C. The zeta potential (ZP) was determined by electrophoretic mobility analysis under the same conditions as DLS measurements, using the Smoluchowski theory. All measurements were made using the Zetasizer Nano ZS90 (Malvern, UK). Formulations were previously diluted at a 1:100 ratio in deionized water for size measurement and 0.9% NaCl for ZP.

Liposomal morphology was analyzed by cryogenic-transmission electron microscopy (Cryo-TEM). LAmB sample was prepared on carbon-coated grids with 1% sodium phosphotungstate for negative staining. Cryo-TEM samples were vitrified (25 °C, ∼100% RH) using a controlled environment vitrification system and imaged with a Tecnai G2–20 FEI SuperTwin (200 kV) at the UFMG Microscopy Center.

#### 2.3.2 Determination of AmB and RA Encapsulation Rate

The analytical method used to measure AmB and RA simultaneously followed the literature.^36–38^ Method validation followed the guidelines outlined in the ANVISA RDC N^o^ 166, 2017.^39^ AmB and RA quantification using high-performance liquid chromatography coupled to a diode array detector (HPLC/DAD) was carried out in an Agilent 1260 series chromatograph (CA, USA). Analyses were conducted using a 1 mL/min flow rate, a Discovery C18 column (250 mm × 4.6 mm; 5 μm), and a mobile phase consisting of acetonitrile: methanol: citric acid solution (4.2 g/L) pH 6 (48:12:40) with gradient for RA elution. The injection volume was 20 μL. AmB and RA maximum absorption wavelengths at 405 and 340 nm, respectively, were chosen for detection.

The encapsulation efficiency (EE_(%)_) was determined by comparing AmB and RA peak areas before liposome filtration (*A*_unf_) and after filtration (*A*_f_) using a polyvinylidene fluoride (PVDF) membrane filter (0.45 μm; Millipore, USA). This filtration eliminates the possible drug crystals formed (free drug). EE_(%)_ was calculated using eq 1.

1 $${\mathrm{EE}}_{\left(\%\right)}=\frac{A_{f}}{A_{\mathrm{unf}}}\cdot 100$$

#### 2.3.3 AmB Aggregation State

The aggregation state of AmB was analyzed using UV/vis and circular dichroism (CD) spectroscopy, as described in the literature.^19^ Different batches of the LAmB-RA formulation (*n* = 5) were diluted in PBS to reach an AmB concentration of 5 μg/mL. For comparison, commercial products AmBisome and Anforicin B (*n* = 1 each) were reconstituted according to the package leaflet, and then further diluted in PBS to 5 μg/mL. Spectral data were collected in triplicate at 25 °C under nitrogen using a Chirascan spectropolarimeter over the 300–450 nm range. The final spectra were corrected by subtracting the PBS baseline. The DC signal was smoothed using a factor of 4. No signal was detected for the Empty liposome.

#### 2.3.4 Small-Angle X-ray Scattering (SAXS) Analysis

SAXS experiments were performed at the EMUSAXS Multiuser Facility at the Institute of Physics of the University of São Paulo, using a XEUSS 2.0 lab system from Xenocs. This instrument has a microfocus source copper Genix3D (λCukα = 1.5418 Å), Fox3D mirror and two sets of scatterless slits. The two-dimensional scattering data were collected with a Dectris-Pilatus 300k detector processed with the Fit2D software package.^40^ The scattering intensity was shown as a function of the reciprocal space momentum transfer modulus, *q* = 4π sin­(θ)/λ, where 2θ is the scattering angle and λ is the wavelength of the radiation. The samples (LAmB-RA, LAmB, LRA and LEmpty at 22 g/L lipid concentration) were placed in homemade reusable sample holders composed of borosilicate capillaries (1.5 mm) glued in stainless steel cases. The holders were closed with rubber caps allowing measurements in vacuum. Data treatment was done with the SuperSAXS software package,^41^ using a buffer solution as blank and water at 20 °C as standard for absolute calibration.^42^ Measurements were made at room temperature (22 °C) with acquisition times of 1800 s. The sample-to-detector distance was 1185 mm, giving an experimental range of 0.009 < *q* < 0.331 Å^–1^. Data analysis was performed using the Gaussian deconvolution method^43^ as described in the Supporting Information.

### 2.4 Hemolytic Activity *In Vitro*

Evaluation of *in vitro* hemolytic activity was carried out as previously described.^36^ To separate red blood cells (RBC), rabbit blood collected in sodium citrate was centrifugated at 3000*g* for 5 min at 4 °C. The supernatant and the buffy coat were discarded, and RBC was washed with PBS pH 7.4 and centrifuged as previously; this procedure was performed twice. Two volumes of RBC were mixed with 11 parts of PBS pH 7.4 to obtain a stock suspension.

Subsequently, 1 mL of the RBC stock suspension was mixed with 1 mL of diluted formulations (LAmB, LRA, LAmB-RA, AmBisome and Anforicin B) in PBS pH 7.4 containing 20, 40, 75, 140, and 250 μg/mL of the AmB equivalent. Each concentration was tested in triplicate. RBC mixed with ultrapure water and PBS were used as positive and negative controls, respectively. Samples were incubated at 37 °C for 3 h in a shaker bath and then centrifuged at 1,700 g for 10 min to separate the supernatant, which stood for 30 min at room temperature to oxidize hemoglobin. Oxygenated hemoglobin absorbance was measured in a spectrophotometer (Evolution 201 UV–visible Spectrophotometer, Thermo Fisher Scientific) at 540 nm. The hemolysis percentage (*H*_(%)_) was calculated using eq 2.

2 $$H_{\left(\%\right)}=\frac{{\mathrm{AB}}_{s}-{\mathrm{AB}}_{0}}{{\mathrm{AB}}_{100}-{\mathrm{AB}}_{0}}\cdot 100$$

Where AB_s_ is the sample absorbance, and AB_100_ and AB_0_ are the absorbance of the positive and negative controls, in that order.

### 2.5 Pharmacokinetic Study

#### 2.5.1 Treatment of Animals and Samples Collection

Female Swiss mice (4–6 weeks old) were randomly divided into three groups: LAmB, LAmB-RA, and AmBisome. The animals received a single dose equivalent to 1 mg/kg of AmB by intravenous injection (IV). At 5 min, 0.5, 1, 4, and 24 h postinjection, blood and liver were collected (*n* = 5 mice per time point). The blood was collected by intracardiac puncture in tubes containing 0.1 M EDTA. Blood was centrifuged at 1400*g* for 10 min, and the plasma samples (supernatant) and livers were collected and frozen at −80 °C until analysis by liquid chromatography–mass spectrometry (LC-MS/MS). This study was approved by the Ethical Committee for Animal Experimentation under protocol number #30494–2021031617294635v1.

#### 2.5.2 Bioanalytical Method for AmB Determination

A bioanalytical method was validated to determine AmB concentrations in plasma and liver, employing LC-MS/MS, as described in detail in the Supporting Information.

#### 2.5.3 Pharmacokinetic Parameters

The model that best fitted the plasma concentration–time data and the pharmacokinetic parameters was a two-compartment open model with IV bolus input, determined using the Rstrip 4.03 computer software. Fitted parameters included the early elimination phase half-life (*t*_1/2α_), the terminal phase half-life (*t*_1/2β_), the area under the blood concentration–time curve, the mean residence time projected to infinity, and the elimination rate constant.

### 2.6 *In Vivo* Assessment of the Therapeutic Efficacy and Immunomodulation of Liposomal Formulations

#### 2.6.1 Cell Culture

For the *in vitro* propagation of *L*. (*L.*) *major* (MHOM/PT/92/CRE26) promastigotes, complete Medium 199 (Sigma, USA) was used, enriched with 10% fetal bovine serum (Gibco, Germany). The promastigotes were maintained in culture until they reached the stationary phase of growth.

#### 2.6.2 Infection of Animals and Treatment Protocols

Female BALB/c mice (4–6 weeks old) were infected subcutaneously at the tail’s base with 100 μL of a suspension containing 1 × 10^7^ *L. major* late-log promastigotes. After lesions development (27 days postinfection), animals were divided into six groups (*n* = 8 for each group, and *n* = 10 for Control), as follows: Group 1–Control (untreated); Group 2–LEmpty; Group 3–LRA (0.25 mg/kg); Group 4–LAmB (5 mg/kg); Group 5–LAmB-RA (5 mg AmB/kg–0.25 mg RA/kg); and Group 6–AmBisome (5 mg/kg). Animals were treated intraperitoneally (IP) (0.2 mL) every 2 days for 18 days. This study was approved by the Ethical Committee for Animal Experimentation under protocol number #30494–2021031617294635v1.

In the CL murine model using *L. amazonensis* (IFLA/BR/1967/PH8), female BALB/c mice (4–6 weeks old) were infected subcutaneously at the tail’s base with amastigotes freshly recovered from infected hamster’s paw, as previously described.^44^ After lesions development (44 days postinfection), animals were divided into five groups of 7 animals each, as follows: Group 1–Control (untreated); Group 2–LEmpty; Group 3–LAmB (5 mg AmB/kg); Group 4–LAmB-RA (5 mg AmB/kg and 0.25 mg RA/kg); and Group 5–AmBisome (5 mg AmB/kg). Animals were treated by IP (0.2 mL) every 2 days for 24 days. This study was approved by UFMG Ethical Committee for Animal Experimentation under the protocol number 331/2022.

#### 2.6.3 Treatment Efficacy

In both models, lesion size was determined with a caliper by obtaining the average diameter traced from one lesion border to another.^36^ The lesion size growth was calculated for each animal based on the difference in the average lesion size during treatment and the first day of treatment. Lesions were photographed for qualitative comparison. Three days after the last dose, the animals were euthanized, the lesion and spleen were collected and the parasite load was analyzed by qPCR, as previously reported.^45,46^ The detailed protocol can be found in the Supporting Information.

#### 2.6.4 Cytokine Profile Determination

In *L. major*-infected mice, a liver fraction (approximately 200 mg) was collected 3 days after the last dose and further macerated and homogenized using a glass rod in 1 mL of lysis buffer containing 50 mM Tris-base pH 7.4, 150 mM NaCl, and 5 mM EDTA. Buffer was supplemented with 1% Nonidet P-40, 1 mM Phenylmethylsulfonyl Fluoride (PMSF), and a protease inhibitor cocktail (P8340, Sigma-Aldrich, USA). Liver homogenates were kept for 30 min on ice, then centrifuged for 10 min at 10,000*g* and 4 °C. Supernatants were collected and stored at −20 °C until the next day for ELISA and bead-based cytokine quantification assay.^47^ Before analysis, samples were centrifuged for 2 min at 10,000*g* at 4 °C to exclude potential debris. IFN-γ quantification was performed using the ELISA Max Standard set (BioLegend, USA). A panel of cytokines and chemokines: transforming growth factor-β (TGF-β1), CCL22, IL-10, IL-6, and granulocyte colony-stimulation factor (G-CSF) was assessed using the LEGENDplex bead-based immunoassay according to the manufacturer’s instructions (BioLegend, USA).

In *L. amazonensis*-infected mice, the spleen was used to assess the immune response. The spleen was macerated and homogenized, and the obtained cell suspension was adjusted at 5 × 10^6^ cells/mL in RPMI supplemented with 10% of fetal bovine serum. Splenocytes were incubated in the absence or presence of different stimuli: Soluble *Leishmania* Antigen (SLA)—50 μg/mL, prepared as reported in the Supporting Information; Concanavalin A (ConA)—10 μg/mL and RA—0.5 nM for 48 h at 37 °C, under 5% CO_2_. The culture medium itself was used as a negative Control. The concentrations of IFN-γ and IL-10 in the cell culture supernatants were performed by ELISA using the OptEIA Mouse Kit (BD Biosciences, USA) according to the manufacturer’s instructions.^48,49^

#### 2.6.5 Toxicity Evaluation

Plasma markers of renal (urea, creatinine) and hepatic (ALT, AST) toxicities were investigated 3 days after the last dose of treatment of *L. amazonensis*-infected mice. Animal blood was collected by puncture of the brachial plexus in tubes containing EDTA 0.1 M immediately after anesthesia with a mixture of ketamine (100 mg/kg) and xylazine (15 mg/kg). After blood collection, kidneys were also collected for histopathological examination as described in the Supporting Information. The kidney sections were evaluated for degenerative changes and inflammatory infiltration. A score of 0 to 8 was set to measure renal alterations, with 0 to 1: absent, 2 and 3: mild, 4 and 5: moderate, 6 and 7: intense, 8: severe. The total score was obtained by adding the degenerative changes and inflammatory infiltrate scores. The animals’ weight was recorded on each day of treatment as a parameter for assessing toxicity in both *L. major* and *L. amazonensis* murine models.

### 2.7 Statistical Analysis

The statistical analyses were performed using GraphPad Prism (version 8). Data normality and variance homogeneity were assessed using the Shapiro–Wilk or Kolmogorov–Smirnov test, and the Brown–Forsythe or *F* test, respectively. One-way ANOVA with Tukey’s, Dunnett’s, or Holm–Šídák’s post-test was used to analyze liposomes’ physicochemical characteristics, *in vitro* hemolytic activity, parasite load, and cytokine profiles in *L. major*-infected mice, and IFN-γ/IL-10 ratio. Nonparametric data were analyzed using the Kruskal–Wallis test with Dunn’s post-test. Two-way ANOVA with appropriate post-tests was used for the pharmacokinetics, lesion size, cytokine levels after stimulation, and animal weight. A 95% confidence level was considered, with significance set at *p*-value (*p)* < 0.05.

## 3 Results

### 3.1 Development of LAmB-RA and Liposomes Physicochemical Characterization

Co-incorporation of RA and AmB in preformed PEGylated liposomes was achieved by exploiting the pH dependence of the solubility of both drugs. While RA and AmB are highly soluble in alkaline solutions, their solubility decreased at neutral pH, favoring their incorporation into the liposomal membrane. Two AmB/RA molar ratios were evaluated: 1:0.30 and 1:0.15. The choice of the best molar ratio was based on the formulation’s visual stability. Despite having suitable particle size distribution after preparation (diameter = 117 ± 3 nm; PdI = 0.15 ± 0.02), the liposome with the highest amount of RA (1:0.30) showed precipitation 2 days after storage at 4 °C.

As depicted in Table 1, control PEGylated liposomes containing only AmB (LAmB), only RA (LRA), or no drug (LEmpty) were prepared for comparison with LAmB-RA (1:0.15 molar ratio), which means a final AmB concentration of 2.9 mg/mL and RA of 0.145 mg/mL. LAmB-RA exhibited a hydrodynamic diameter of 123 ± 6 nm and a PdI of 0.11 ± 0.02, values comparable to those of LAmB (128 ± 4 and 0.10 ± 0.03), as shown in the intensity plots in Figure S1. In contrast, LRA and LEmpty showed significantly smaller vesicle diameters (*p* < 0.0001). As no increase in size was observed between LRA and LEmpty, the vesicle enlargement was mainly attributed to AmB.

**Table 1:** Liposomes’ Physicochemical Characteristics^b^

formulation	size (nm)	PdI	ZP (mV)	AmB EE_(%)_	RA EE_(%)_
LEmpty	92 ± 9	0.09 ± 0.05	–4.9 ± 0.5	-	-
LRA	89 ± 9	0.13 ± 0.04	–5.5 ± 1.5	-	96.8 ± 5.0
LAmB	128 ± 4^a^	0.10 ± 0.03	–4.2 ± 1.1	96.8 ± 5.9	-
LAmB-RA	123 ± 6^a^	0.11 ± 0.02	–5.3 ± 1.2	97.1 ± 4.4	94.7 ± 5.2

a Significant difference compared to LEmpty and LRA (*p* < 0.0001) according to the one-way ANOVA followed by Tukey’s multiple comparison post-test. EE_(%)_–Encapsulation efficiency; Size—hydrodynamic diameter; PdI–Polydispersity index; ZP–Zeta potential.

b Data are mean ± SD from at least 8 independent batches.

PdI values close to 0.1 were observed for all liposomal formulations (Table 1), indicating monodisperse systems. The evaluation of the ZP, in turn, reflects particles’ electrical potential, i.e., their surface charge.^50^ Determination of the particle ZP showed similar slightly negative values around −5.0 mV for the different formulations (Table 1). Despite the low surface charge, liposome aggregation can be prevented by steric hindrance due to vesicles PEGylation. Table 1 also shows that encapsulation rate in LAmB-RA was 97.1 ± 4.4% for AmB and 94.7 ± 5.2% for RA. Similar values of drug encapsulation rates were found in the formulations containing only AmB and RA.

As shown in Figure 1A, cryo-TEM analysis of LAmB confirmed the presence of unilamellar vesicles. SAXS analyses of LAmB and LAmB-RA formulations were also carried out to investigate the possible effect of RA incorporation on the vesicle and bilayer structures. The overall shape of the obtained scattering intensity was characteristic of lipid bilayers^43,51–53^ and the theoretical model described very well the experimental data for both samples, as shown in Figure 1B.

**Figure 1:**
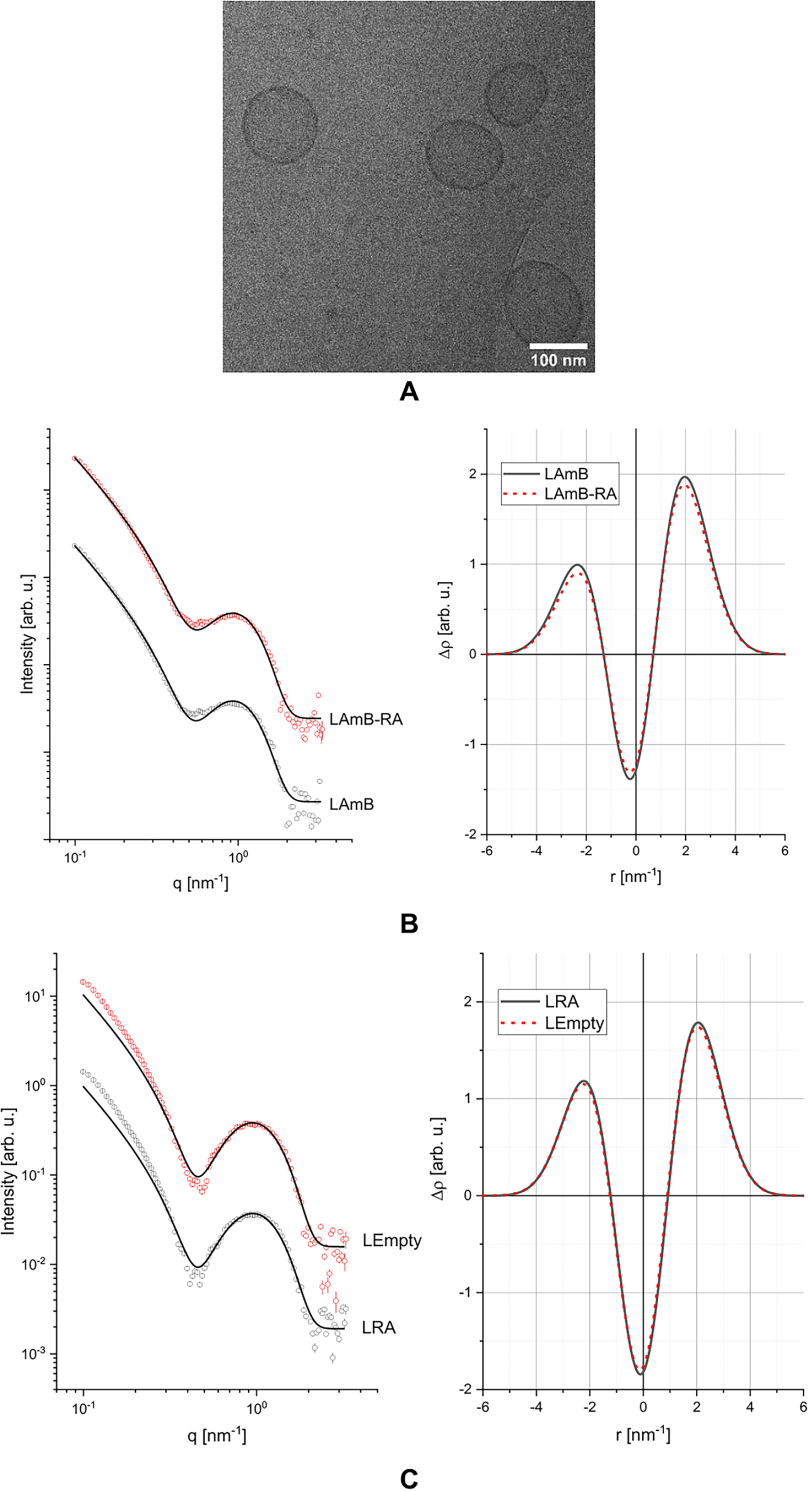
(A) Cryo-TEM image of the LAmB formulation. (B, C) SAXS profiles with experimental data (symbols) and corresponding theoretical fits (solid lines) and electron density profile (EDP) derived using the Gaussian Deconvolution method.

The lack of difference in the electronic density profile (EPR) between LAmB and LAmB-RA indicates that RA did not induce significant structural changes in the liposomal structure (Figure 1B). The SAXS profile is also consistent with the lack of appearance of distinct nanoassemblies due to RA. Interestingly, the liposomal formulations without AmB (LEmpty and LRA) showed deeper minima in the SAXS curves (Figure 1C), which is an indication of more symmetric bilayers.^43^ The more asymmetric bilayer in LAmB and LAmB-RA may be related to the mode of incorporation of AmB into preformed empty liposomes, which may result in the predominant localization of AmB in the outer layer of the liposome membrane.

UV–vis spectroscopy is a sensitive method for assessing AmB aggregation. As shown in Figure S2A, both LAmB and LAmB-RA displayed similar absorption spectra, featuring a strong band at 329 nm and three less intense bands at 364, 388, and 417 nm. AmBisome showed a comparable pattern but with a blue-shifted main peak at 323 nm, indicating a superaggregated AmB form in the liposomal membrane.^54^ Anforicin B also exhibited a prominent peak at 330 nm, associated with aggregated AmB species.^55,19^

Figure S2B,C reveal that LAmB and LAmB-RA exhibited similar CD spectra, featuring a positive peak at 327 nm and a negative peak at 342 nm. LAmB showed stronger signal intensity than LAmB-RA, indicating that RA may influence AmB’s aggregation or structural arrangement within the liposome—more clearly seen in the inset of Figure S2C. The CD spectrum of AmBisome reflected a more aggregated AmB form, with a pronounced couplet signal: a positive Cotton effect at 323 nm and a negative one at 334 nm. Anforicin B also displayed a strong doublet, more intense than those of the other formulations. These spectral differences suggest that LAmB and LAmB-RA contain AmB in a less aggregated state compared to AmBisome.

### 3.2 *In Vitro* Hemolytic Activity

Liposome formulations LAmB, LAmB-RA, and LRA were further characterized regarding *in vitro* hemolytic activity. Figure 2A illustrates the hemolytic activity across various AmB concentrations. For each liposomal AmB formulation, the hemolytic activity increased with AmB concentration. Although LAmB-RA and AmBisome did not differ significantly at 140 μg/mL, lower toxicity was observed for AmBisome (14.9 ± 0.8%), followed by LAmB-RA (21.6 ± 0.2%) and LAmB (28.0 ± 0.2%) at the highest AmB concentration (250 μg/mL) (Figure 2B). It is worth noting that adding RA reduced significantly (*p* < 0.0001) the hemolysis percentage (LAmB vs. LAmB-RA). In contrast, Anforicin B caused 99.7 ± 0.2% hemolysis even at the lowest AmB content (20 μg/mL) (Figure 2A).

**Figure 2:**
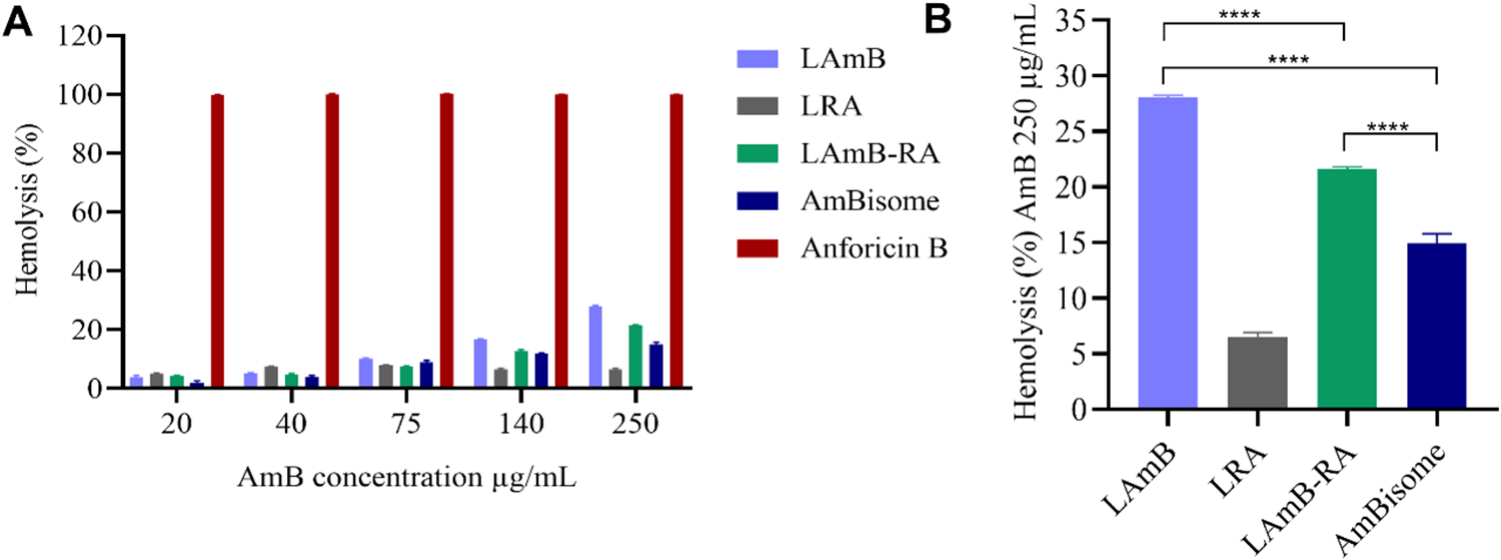
*In vitro* hemolytic activity caused by different formulations of AmB: (A) AmB formulations in different concentrations; (B) comparison between AmB formulations without Anforicin B at the highest AmB concentration (250 μg/mL) for better visualization. Data are mean ± SD (*n* = 3) and analyzed through one-way ANOVA followed by Tukey’s multiple comparison post-test. ****Significant differences observed between the AmB-containing formulations (*p* < 0.0001).

### 3.3 Pharmacokinetic Study

Figure 3A displays the concentration of AmB in mouse plasma as a function of time after IV bolus administration of liposomal AmB formulations at 1 mg AmB/kg. A two-compartment open model with IV bolus input best fitted experimental plasma concentration–time data. Table 2 shows the calculated pharmacokinetic parameters. LAmB (0.38 h) and LAmB-RA (0.44 h) showed early elimination half-life (*t*_1/2α_) values comparable to the commercial liposomal formulation (0.60 h). On the other hand, both PEGylated formulations exhibited a higher terminal phase half-life (*t*_1/2β_), greater areas under the concentration–time curve (AUC), and mean residence times of AmB, compared to AmBisome. Notably, LAmB showed the highest AUC_0–inf_ (54,541 ng/mL·h), around 2 times higher than that of AmBisome (23,733 ng/mL·h).

**Figure 3:**
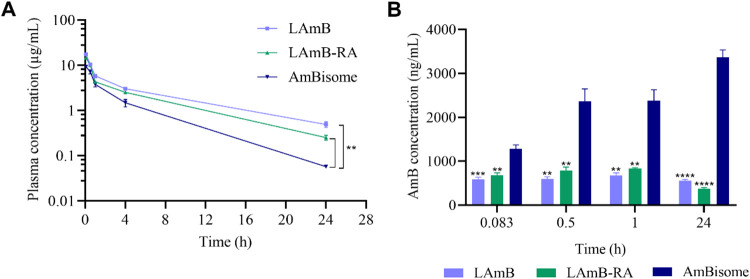
Pharmacokinetic analysis of AmB in (A) plasma and (B) liver of Swiss mice after intravenous administration of LAmB, LAmB-RA, or AmBisome at 1 mg/kg. Data are mean ± SEM (*n* = 5). AmB plasma concentration is presented as logarithmic concentration–time curves. Plasma concentrations at 24 h were analyzed through two-way ANOVA followed by Dunnett’s multiple comparison post-test, showing significant differences compared to AmBisome group (**p < 0.01). AmB concentrations in the liver showed significant differences compared to AmBisome at each time point (**p < 0.01, ***p < 0.001, ****p < 0.0001, Two-Way ANOVA).

**Table 2:** Pharmacokinetic Parameters of LAmB, LAmB-RA, and AmBisome Formulations^a^

	groups		
parameters	LAmB	LAmB-RA	AmBisome
*C*_max_ (ng/mL)	18,656	15,648	9,658
*t*_1/2α_ (h)	0.38	0.44	0.60
*t*_1/2β_ (h)	8.80	9.13	6.54
AUC_0–*t*_ (ng/mL·h)	47,562	37,237	22,402
AUC_0–inf_ (ng/mL·h)	54,541	42,830	23,733
MRT_0–inf_ (h)	10.85	10.76	6.98
*k*_el_ [1] (h^–1^)	0.079 ± 0.036	0.076 ± 0.063	0.106 ± 0.162
*k*_el_ [2] (h^–1^)	1.810 ± 0.220	1.579 ± 0.283	1.157 ± 0.341

a *C*_max_–maximum AmB concentration in plasma; *t*_1/2α_–early elimination phase half-life; *t*_1/2α_–terminal phase half-life; AUC–area under the concentration–time curve; MRT–mean residence time; *k*_el_–elimination rate constant expressed as mean ± SD (*n* = 5).

Figure 3B shows the concentration of AmB in mouse liver at different time intervals after administration. Interestingly, the AmBisome group showed the highest AmB level in the liver compared to the other two formulations. It is also noteworthy that AmB levels in the liver increased over time in the AmBisome group, which was not observed in the LAmB and LAmB-RA groups.

### 3.4 Antileishmanial Efficacy and Immune Response in Murine Models of CL

LAmB-RA and LAmB were further evaluated for their therapeutic efficacies in two different murine models of CL, in comparison to nontreated Control and groups receiving empty liposomes or AmBisome. The impact of treatments was assessed on the lesion size growth and the parasite load in the lesion or spleen, as well as cytokine production in the liver or spleen.

#### 3.4.1 CL Caused by *L. major*

A significant reduction in lesion size growth (*p* < 0.05) compared to the Control (Figure 4A,4B) was observed in all groups receiving liposomal AmB formulations. In particular, *L. major*-infected mice receiving PEGylated liposomal AmB, with or without RA, led to smaller lesions at the end of treatment compared to the first day. This result was not observed in the AmBisome group, where only the progression of the lesion size was slowed down compared to the Control. Lesion photographs for qualitative comparison were taken on the last day of treatment, as seen in Figure S3A. Interestingly, LRA did not reduce lesion growth or parasite load compared to the Control: no beneficial or harmful effects were observed. Possibly, at this dose, RA alone had no potent antileishmanial effect, and a combination with AmB was necessary. RA benefit of the combination with AmB was observed in reducing the parasite load in the lesion (Figure 4C). LAmB-RA was the group with the greatest decrease in parasite load compared to the Control (99%), followed by LAmB (94%). It should be noted that there was no substantial reduction in the parasite load when animals were treated with AmBisome (51%).

**Figure 4:**
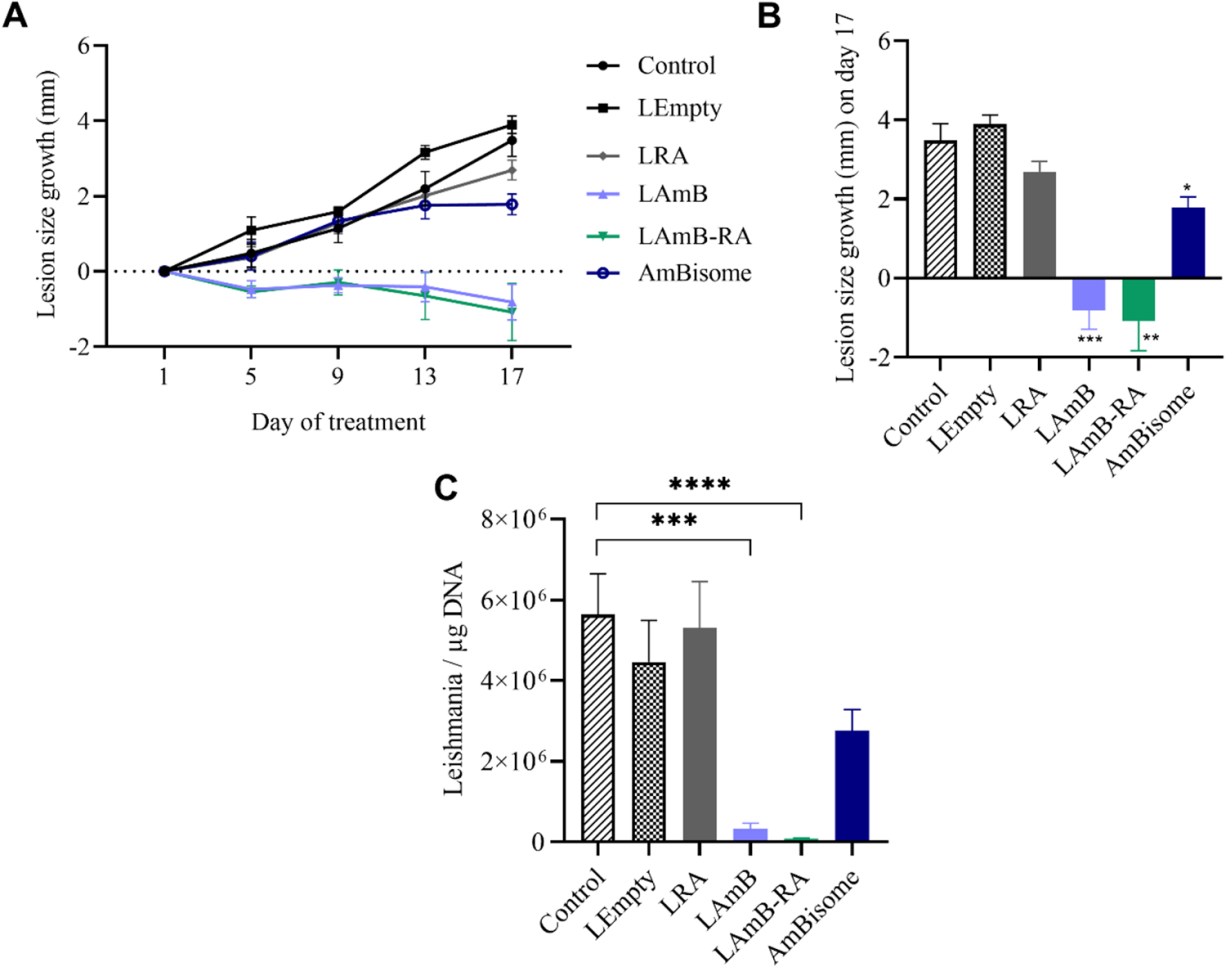
Therapeutic efficacy of different liposomal AmB formulations by IP route in *L. major*-infected mice, on the lesion size growth (A, B) and parasite load in the lesion (C). *L. major*-infected BALB/c mice were treated for 18 days every 2 days. Lesion size growth was calculated for each animal based on the difference in the average lesion size during treatment and the 1st day of treatment. The lesion size was measured in the course (A) and at the end of treatment (B). The parasite load in the lesion was determined by qPCR (C). (B) Data are mean ± SEM (*n* = 8 for each treatment group, and *n* = 10 for the Control group) and analyzed through repeated measures of two-way ANOVA followed by Tukey’s multiple comparison post-test. Significant differences were observed compared to the Control group (*p < 0.05, **p < 0.01, ***p < 0.001). (C) Data are mean ± SEM and analyzed through one-way ANOVA followed by Tukey’s multiple comparison post-test. Significant differences were observed compared to the Control group (***p < 0.001, ****p < 0.0001).

Cytokines and chemokines were assessed directly in the supernatant obtained from liver homogenates. A significant increase in IFN-γ levels was observed only for the LRA (474.63 ± 56.29 pg/mL) and AmBisome (612.45 ± 47.65 pg/mL) groups (Figure S4A). When looking at IL-10 levels, on the other hand, there was a trend toward higher levels in the group treated with LAmB, but none of the treatments led to a significant increase compared to the Control (Figure S4B). Thus, evaluating the IFN-γ/IL-10 ratio indicated a significantly lower ratio in LAmB compared to AmBisome and an intermediate ratio in the case of LAmB-RA (Figure S4C).

A panel of cytokines and chemokines involved in anti-inflammatory and tissue-repairing activities was also evaluated. M2 macrophages decrease immune reactions and promote tissue repair by releasing different factors, such as Free Active TGF-β1, CCL22 (MDC), IL-6, and G-CSF. These cytokines and chemokines counteract the immunopathology caused by pro-inflammatory cytokines and, depending on the disease course, are also responsible for maintaining parasites in the infection site and chronicity.^57^ A significant increase in TGF-β1 was observed for LEmpty compared to LAmB (*p* < 0.05), but no differences was observed compared to the Control group (Figure S4D). Furthermore, a reduction in CCL22 in LAmB-RA compared to AmBisome (Figure S4E) was observed. The chemokine CCL22 is known to recruit regulatory T cells (Tregs) to the site of infection, suppressing inflammatory immune responses.^58^ However, no significant difference was found for this chemokine compared to the Control group. Similarly, there were no significant differences between the groups for IL-6 or G-CSF levels (Figure S4F,G).

Mice weight was monitored as an indirect parameter of toxicity. Even though the animals receiving AmB fluctuated in weight during the treatment, no significant weight loss was observed for any treatments comparing the animals’ weight at the beginning and the end of treatment (Figure S3B).

#### 3.4.2 CL Caused by *L. amazonensis*

Evaluation of lesion size growth at the end of treatment (Figure 5A,5B) showed a marked reduction (*p* < 0.05) in mice treated with LAmB-RA (1.1 ± 0.7 mm) compared to untreated Control animals (3.3 ± 1.4 mm). Notably, LAmB-RA significantly reduced the animal’s lesion size growth compared to AmBisome (2.5 ± 0.8 mm; *p* < 0.05). All groups treated with liposomal AmB formulations also exhibited significantly less pronounced lesion size growth, when compared to LEmpty group (4.0 ± 0.5 mm; *p* < 0.05). The group receiving LRA alone was not included in this study, as LRA did not promote any beneficial or harmful effects in the *L. major* model (Figure 4). Lesions photographs for qualitative comparison were taken on the 23rd day of treatment, as seen in Figure S5A.

**Figure 5:**
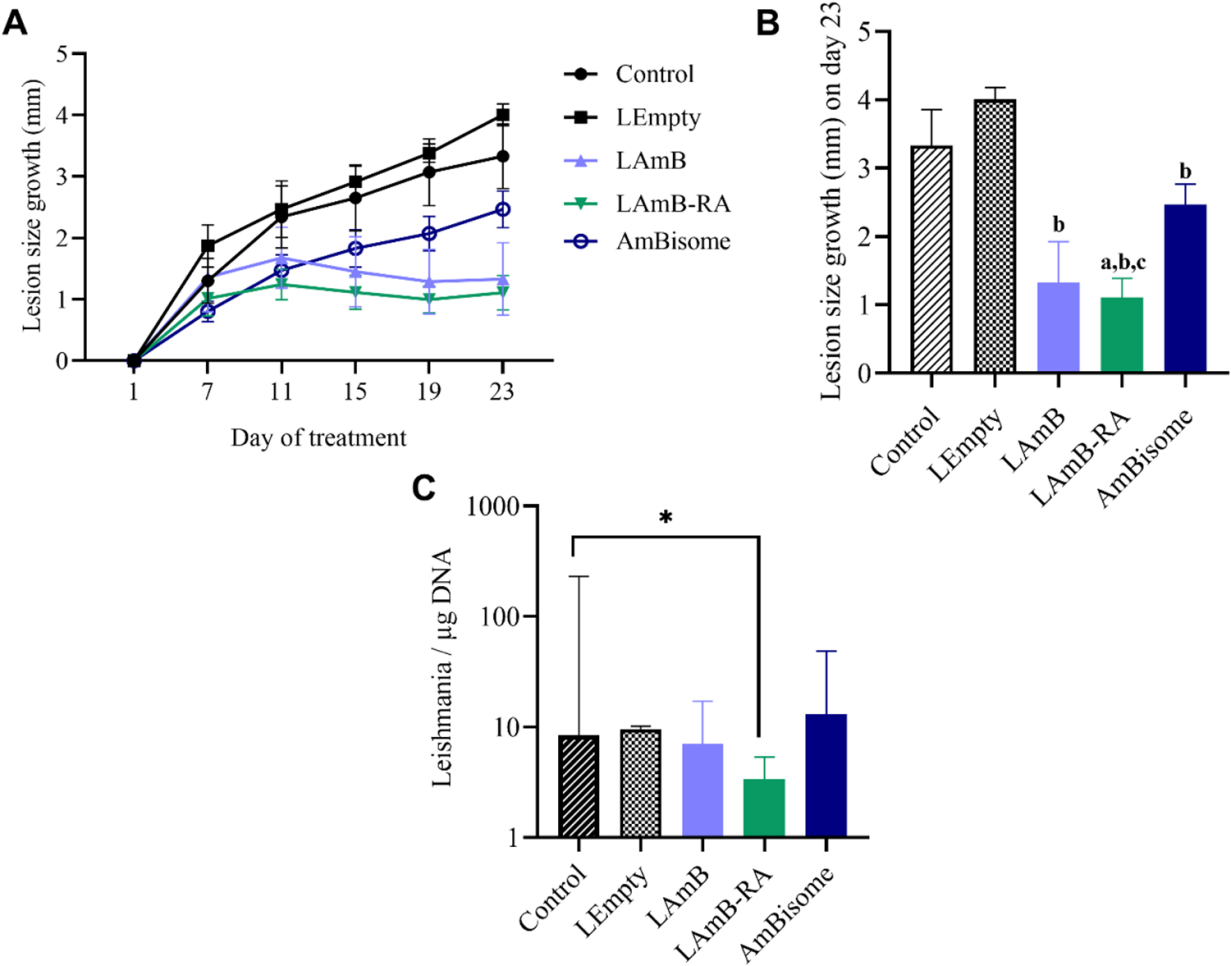
Therapeutic efficacy of different liposomal AmB formulations by IP route in *L. amazonensis*-infected mice, on the lesion size growth (A, B) and parasite load in the spleen (C). *L. amazonensis*-infected BALB/c mice were treated for 24 days every 2 days. Lesion size growth was calculated for each animal based on the difference in the average lesion size during treatment and the 1st day of treatment. The lesion size was measured in the course (A) and at the end of treatment (B). The parasite load in the spleen was determined by qPCR (C). (B): data are mean ± SEM (*n* = 7) and analyzed through repeated measures of two-way ANOVA followed by Tukey’s multiple comparison post-test. ^a^Significant differences observed compared to the Control group; ^b^Significant differences observed compared to LEmpty; ^c^Significant differences compared to AmBisome. (C): Data are medians +95% confidence intervals, analyzed through Kruskal–Wallis followed by Dunn’s multiple comparison post-test. Significant differences were observed compared to the Control group (*p < 0.05).

As shown in Figure 6A, when splenocytes recovered from animals were stimulated with culture medium, there was a trend toward greater IFN-γ production in LAmB-RA group compared to the Control (*p* < 0.1). Stimulation with RA, in turn, significantly increased (*p* < 0.05) IFN-γ production in all groups (Control, LEmpty, LAmB, and AmBisome) compared to the culture medium. This increase was not significant in the LAmB-RA group. Similarly, stimulation with SLA led to a significant increase in IFN-γ levels in all groups compared to culture medium. The cellular response to stimulation with the pathological agent can explain this phenomenon. Notably, LAmB-RA was the only group with significantly higher production of IFN-γ in response to *Leishmania* antigen stimulation, compared to the Control group (*p* < 0.001). Splenocytes were also stimulated with ConA, a positive control of activation. Regarding IL-10 production (Figure 6B), LAmB group showed the highest IL-10 levels compared to the Control group for the different stimuli (RA, SLA, and ConA). Introducing RA into the LAmB significantly reduced IL-10 secretion to levels comparable to those of AmBisome.

**Figure 6:**
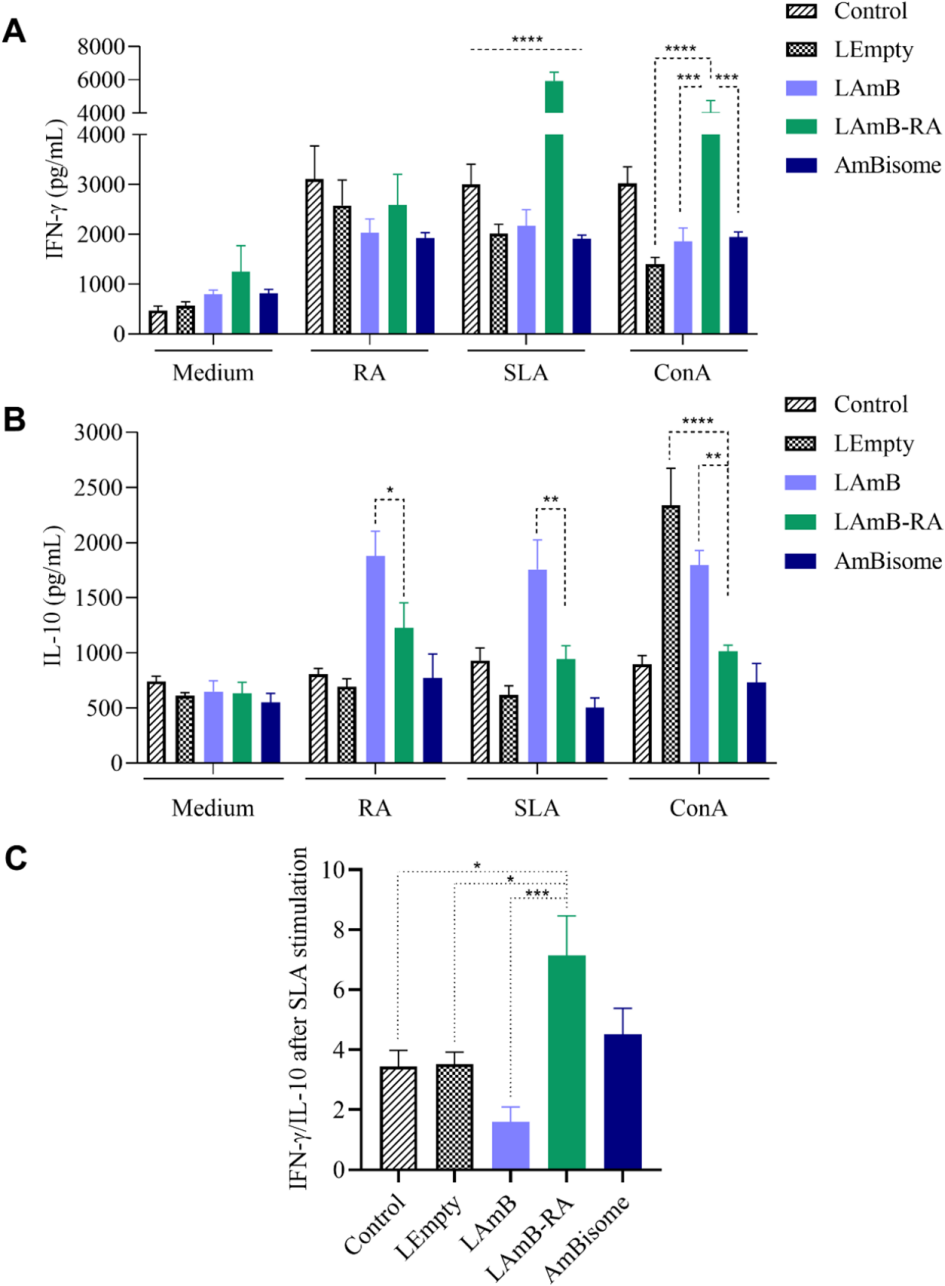
Impact of treatment of *L. amazonensis*-infected mice with different liposomal AmB formulations by IP route on cytokine profile in splenocytes culture. (A) IFN-γ, (B) IL-10, and (C) IFN-γ/IL-10 ratio. *L. amazonensis*-infected BALB/c mice were treated for 24 days every 2 days. Animals were euthanized 3 days after the last dose, the spleens were collected, and splenocytes were subjected to different stimuli: medium, retinoic acid (RA), Soluble Leishmania Antigen (SLA), and Concanavalin A (ConA) before cytokines evaluation by ELISA. Data are mean ± SEM (*n* = 7). (A, B) were analyzed through two-way ANOVA followed by Tukey’s multiple comparison post-test. (C) IFN-γ/IL-10 ratio was analyzed through one-way ANOVA followed by Tukey’s multiple comparison post-test. Significant differences were observed compared to the LAmB-RA group (*p < 0.05, **p < 0.01, ***p < 0.001, ****p < 0.0001).

Regarding the IFN-γ/IL-10 ratio after SLA stimulation, the inclusion of RA in liposomal AmB led to a significantly higher IFN-γ/IL-10 ratio, showing a clear shift toward a Th1-type response (Figure 6C). It should be noted that BALB/c mice are highly susceptible to CL, with this susceptibility linked to a genetic inability to produce IL-12, an essential cytokine for Th1 cell differentiation. This immune impairment causes progressive skin lesions and visceral invasion.^59^ Thus, this fact reinforces that the production of Th1-type cytokines is directly related to animal treatment. Since the parasite can visceralize in BALB/c mice infected with *L. amazonensis*, the parasite load was assessed in the spleen in this model. In line with cytokine production, only the group treated with LAmB-RA showed a significant reduction (60%) in parasite load, when compared to the Control group (Figure 5C).

Assessments of biochemical markers of renal and hepatic functions were performed in *L. amazonensis*-infected mice (Table 3). This analysis showed an increase in creatinine in animals treated with liposomal AmB formulations: LAmB-RA (0.33 ± 0.06 mg/dL), AmBisome (0.33 ± 0.03 mg/dL), and LAmB (0.38 ± 0.07 mg/dL), the last one leading to a more significant increase compared to the Control (0.25 ± 0.04 mg/dL), evidencing some renal toxicity (Table 3). All other markers (ALT, ASAT, urea) did not show significant differences between experimental groups.

**Table 3:** Biochemical Markers of Renal and Hepatic Functions in *L. amazonensis*-Infected BALB/c Mice after Treatment with Different Liposome Formulations of AmB^a^

	groups				
parameters	Control	LEmpty	LAmB	LAmB-RA	AmBisome
creatinine (mg/dL)	0.25 ± 0.04	0.27 ± 0.04	0.38 ± 0.07***	0.33 ± 0.06*	0.33 ± 0.03*
urea (mg/dL)	54.6 ± 7.8	51.6 ± 8.9	57.6 ± 16.3	59.6 ± 9.6	64.6 ± 8.8
ALT (U/L)	30.5 ± 11.4	32.2 ± 4.7	27.9 ± 8.2	28.7 ± 5.3	29.9 ± 3.8
AST (U/L)	104.5 ± 23.1	110.1 ± 31.9	87.5 ± 29.4	87.0 ± 18.0	113.0 ± 25.8

a Data are mean ± SD (*n* = 7). Significant differences were observed compared to the Control group (*p < 0.05, ***p < 0.001). ALT–alanine aminotransferase; AST–aspartate aminotransferase.

Nevertheless, histopathological examination of the kidneys of *L. amazonensis*-infected mice revealed low to mild renal alterations in all the groups that received AmB. In all the tissues evaluated, the largest area corresponded to healthy areas, but at specific points, such as those indicated in Figure 7, there were tissue alterations as described. The main alterations observed were hyaline cylinders, indicating protein accumulation, pyknotic nuclei typical of cell death, and the presence of mononuclear leukocytes and neutrophils as inflammatory infiltrates. Notably, none of the groups had severe toxicity.

**Figure 7:**
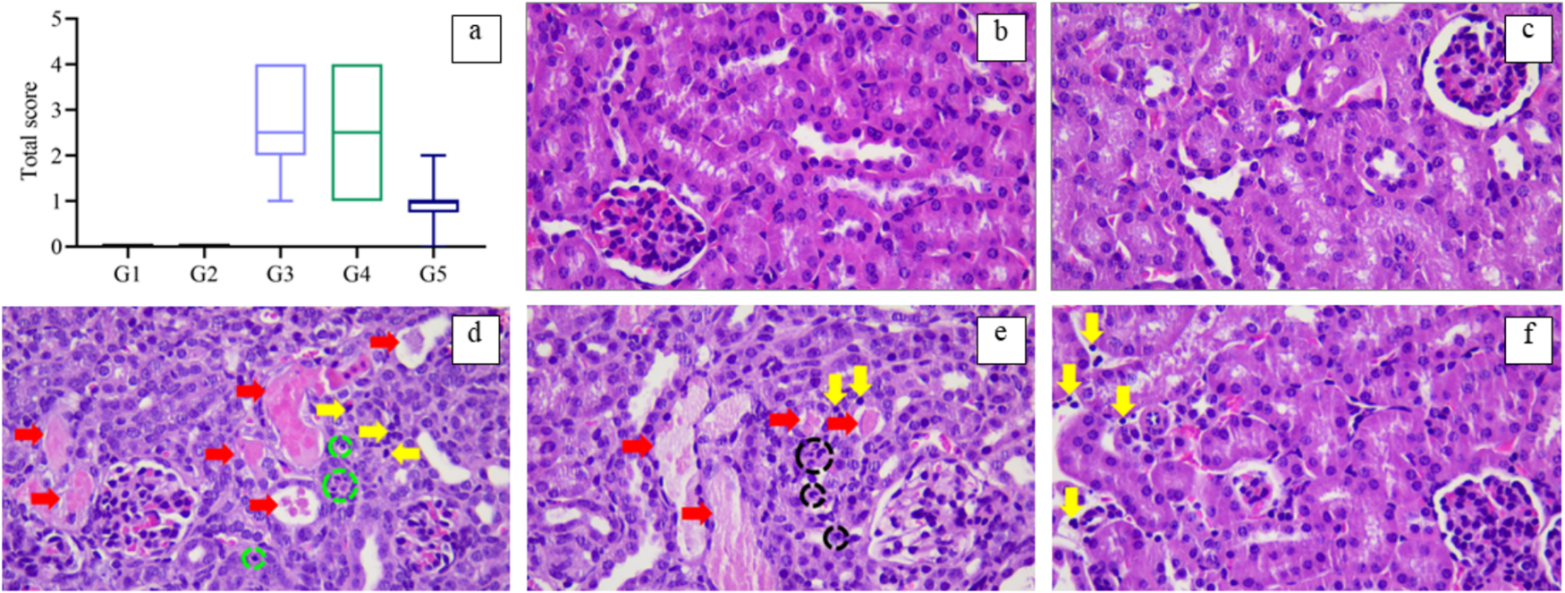
Impact of treatment of *L. amazonensis*-infected mice with different liposomal AmB formulations by IP route on kidney histopathology. (a) A score of 0 to 8 was set to measure renal alterations, with 0 to 1: absent, 2 and 3: mild, 4 and 5: moderate, 6 and 7: intense, 8: severe. G1 = untreated; G2 = LEmpty; G3 = LAmB; G4 = LAmB-RA; G5 = AmBisome. The total score was obtained by adding the degenerative changes and inflammatory infiltrate scores. Data are the median with range (*n* = 7). (b–f) Kidney histological sections of mice are either Control (b), treated with LEmpty (c), LAmB (d), LAmB-RA (e), or AmBisome (f). Yellow arrows indicate mononuclear leukocytes, red arrows represent hyaline cylinders, green circles indicate pyknotic nuclei (cell death), and black circles show neutrophils.

Despite the significant weight fluctuations of animals during treatment, no significant weight loss was observed (Figure S5B) when comparing the weights at the beginning and the end of treatment.

## 4 Discussion

In this study, a novel PEGylated liposomal formulation designed to combine the antileishmanial efficacy of AmB with the immunomodulatory properties of RA^10,23^ was developed and characterized. First, our work successfully addressed the challenge of coincorporating two amphiphilic active agents into preformed PEGylated liposomes, controlling their solubility and incorporation into the liposomal membrane, through precise pH and temperature changes. The formulation exhibited a hydrodynamic diameter of about 125 nm, which is within the range recommended by the United States Pharmacopeia for parenteral administration (≤500 nm).^60,61^ This size minimizes the risks of microvascular obstruction.^62^ Moreover, it favors prolonged circulation.^63,50^ A size increase was noted with AmB incorporation in liposomes, which aligns with prior reports that high drug-to-lipid ratios enlarge liposomes^64^ and decrease membrane deformability.^65^

All formulations showed low polydispersity index values (PdI < 0.2), indicating uniform particle populations, an important parameter for intravenous administration.^66^ The measured ZP values were slightly negative across the different formulations, which could suggest limited electrostatic repulsion. As reported in the literature, coating nanoparticles with hydrated hydrophilic polymers such as PEG can shield the native surface charge, leading to an apparent reduction in ZP.^67,68^ However, in these systems, a low ZP value does not necessarily indicate poor stability, as steric repulsion remains effective.^67^ Importantly, a high EE_(%)_ was achieved for both AmB and RA (>90%). The UV–vis and CD spectra of LAmB and LAmB-RA are consistent with a less aggregated state of AmB, compared to commercial formulations (Anforicin B and AmBisome). Although the SAXS data indicates that RA did not induce major structural rearrangements in the liposomal bilayer, comparison of CD spectra between LAmB-RA and LAmB revealed slightly different aggregated states of AmB. The RA-induced change in the aggregation state of AmB may be due to direct interaction of RA with AmB aggregates or to RA’s influence on membrane packing or fluidity. Although subtle, such variations can directly influence drug release kinetics from liposomes and modulate AmB bioavailability,^69^ which may ultimately impact therapeutic efficacy.

*In vitro* hemolysis assay allowed for an indirect assessment of AmB release, in addition to providing insights into formulation toxicity. It is expected that the greater the AmB availability for interaction with red blood cells, the higher the hemolytic activity. Results revealed lower hemolytic activity for liposomal formulations than the micellar formulation Anforicin B, which is known to promote hemolysis due to rapid AmB release and sodium deoxycholate.^12^ Our findings also indirectly suggest a more rapid release of AmB from LAmB and LAmB-RA than from AmBisome due to a higher hemolysis percentage. This may be attributed to the lower aggregation state of AmB in our liposomal AmB formulations.^69^ Interestingly, adding RA to LAmB formulation significantly reduced hemolysis (*p* < 0.0001).

This protective effect of RA incorporation may arise from the shift in AmB aggregation state toward a less aggregated form, as supported by CD spectroscopy data and previous reports showing that monomeric AmB exhibits lower affinity for cholesterol-rich membranes, thereby reducing damage to mammalian cells while maintaining antiparasitic efficacy.^69^ From this perspective, future studies should investigate how the presence of RA affects AmB release kinetics, using *in vitro* methods such as dialysis or ultrafiltration, commonly applied to liposomal formulations.

Although rabbit RBC are widely used in hemolysis assays due to their availability and sensitivity, interspecies differences in membrane composition and mechanical properties may influence the hemolytic response. Therefore, results should be interpreted with caution, and additional assays using human RBC are desirable to better extrapolate these findings to clinical settings.

A significant difference between LAmB and LAmB-RA formulations and AmBisome is that in-house formulations are PEGylated. Pharmacokinetic data confirmed that PEGylated liposomes exhibited increased *C*_max_, prolonged mean residence time, and reduced hepatic AmB accumulation to approximately 5% of the administered dose at 24 h, compared to 34% for AmBisome. These findings align with the already-known effect of PEGylation on the stealthiness of nanoparticles. However, few studies evaluated the pharmacokinetics of PEGylated liposomes carrying AmB.

Van Etten et al.^71,72^ showed that including DSPE-PEG_1900_ into AmB-liposomes resulted in a prolonged blood residence time compared to non-PEGylated liposomes after a single IV injection in mice. These authors observed a relatively high hepatosplenic uptake (34%–43%) of these liposomes at 24 h after administration. Jung et al.^73^ also reported a positive impact in AUC, *C*_max_, and *t*_1/2_ after lipid nanoparticle PEGylation. Interestingly, both works correlated their results with greater efficacy on fungal infections. Moreover, Kohno et al.^74^ showed higher plasma concentrations of AmB after mice treatment with AmB-PEGylated liposomes, compared to AmBisome and lower AmB concentration in the liver. This result was confirmed by Otsubo et al.^75^

Regarding AmBisome, our results align with published data showing a peak of AmB plasma concentration at an early stage and concentration decay close to zero 24 h after administration. In addition, an increase in liver concentration was also observed over time with this commercial formulation of AmB.^76^ Iman and colleagues^77^ also found a maximum concentration of AmB in mice livers at 24 h following AmBisome administration. The liver was also the organ with the highest AmB levels after autopsy in humans treated with AmBisome.^78^

It is known that AmB pharmacokinetics parameters are influenced by different factors, such as animal strain,^79^ gender,^80^ formulation,^81,82^ and dose.^76^ After administration, AmB may coexist in free, protein-bound, and liposome-associated forms, each with distinct pharmacokinetic profile. A complete characterization of AmB concentration in each pool could be considered in future works.

Efficacy and toxicity studies were carried out in two different murine models of CL using an old-world *Leishmania* species, *L. major*, or a new-world, *L. amazonensis*. In the *L. major* model, treatment with LAmB-RA significantly reduced lesion size growth and parasite burden compared to the Control. It should be noted that AmBisome was less effective than LAmB-RA and LAmB in controlling the lesion size growth and did not promote a substantial reduction in the parasite load, despite a marked increase in IFN-γ level in the liver. These effects are consistent with the pharmacokinetic data of AmBisome, which support the high liver targeting of conventional liposomes, the short mean residence time of AmB in plasma, and, potentially, lower drug accumulation in the skin lesion compared to LAmB and LAmB-RA treatments. Conventional liposomes target visceral organs, especially the liver and spleen, where they are efficiently taken up by phagocytes. This may help explain the clinical success of AmBisome in VL and its variable and often limited efficacy in CL.

One should keep in mind that the cytokine profile obtained from the liver may be different from the profile found in the lesion, as shown by Carvalho et al.^83^ Furthermore, evaluating the amount of cytokine in the organ strongly depends on the disease’s progression and the animal’s immune profile at euthanasia. Thus, our cytokine data should be interpreted with caution, as they may not provide a complete picture of the immune response during the disease.

In the second infection model, LAmB-RA, LAmB, and AmBisome showed a similar efficacy profile as that observed in *L. major*, with a greater ability of LAmB-RA to reduce the lesion size growth compared to Controls and AmBisome. Interestingly, in this infection model, the treatments promoted a slowdown of the lesion growth but not a reduction in the lesion size as observed in the *L. major* model. This outcome could indicate a different drug sensitivity between these species. It is well-known that the *L. amazonensis* species is related to the anergic pole without a specific cellular response to *Leishmania* antigens, leading to diffuse cutaneous leishmaniasis (DCL). In this manifestation, marked parasite proliferation and infection dissemination are observed, with high resistance to treatment and frequent relapses.^5,84^

In this murine model, the immune response was evaluated specifically in the spleen after stimulation with *Leishmania* antigen. Notably, LAmB-RA is the only group with significantly higher IFN-γ production than the Control group (*p* < 0.001). The differences observed in the cytokine profile between the two CL models could be due to the organ since the spleen, a well-established lymphoid organ, may better reflect systemic immune modulation than the liver. Also, different types of immune responses can be observed during different *Leishmania spp*. infections.^6,84^

As *Leishmania* is an intracellular parasite, one of the physiological mechanisms for its elimination by the body is immune cells activation, which is done through cytokine production. Activated macrophages can eliminate the parasite by producing reactive oxygen species.^5,6^ The increased IFN-γ production observed supports RA’s role in enhancing specific Th1-type immune responses. This shift toward a Th1-type response was also observed in recent studies.^10,22–24^ In line with cytokine production, a significant reduction in parasite load was observed in the spleen for the LAmB-RA group (60%), demonstrating the efficacy of the therapy systemically. On the other hand, only a 17% reduction was achieved by LAmB, and no reduction was observed for AmBisome.

Despite the reduction in parasite load and lesion growth in both models, complete wound healing was not observed, likely because this process extends beyond the treatment period. Wound healing in CL involves complex steps such as re-epithelialization, modulation of inflammation, and tissue remodeling. Studies have shown that chronic inflammatory responses in CL lesions can delay healing and contribute to scarring. In this context, the dynamic interplay between pro- and anti-inflammatory cytokines, including IL-10 and TGF-β, as well as the persistence of parasites in the tissue, may influence this process.^86^ Although specific wound healing markers were not evaluated in this study, tissue regeneration through RA mediated immunomodulation, including stimulation of collagen type I synthesis and fibroblast proliferation, remains a potential benefit.^30,87^

To evaluate the potential toxicity of the treatment, creatinine and urea were investigated as serum markers of the renal function and hispathological evaluations were carried out. The slight increase in serum creatinine and changes in histopathological score, in the groups receiving liposomal AmB formulations are indicative of mild renal toxicity. Nephrotoxicity is a known adverse effect of AmB,^12^ affecting over 40% of patients.^88^

This study has some limitations that must be considered. The pharmacokinetics of AmB, as well as the cytokine profile, were not evaluated in the skin, which is the primary site of action in CL. Additionally, the RA dose tested was fixed, preventing conclusions regarding dose–response relationships. For these reasons, future studies should investigate the pharmacokinetics of AmB in the skin to better correlate local drug levels with therapeutic outcomes. Moreover, immune responses could be evaluated directly at lesion site, and the effect of higher RA doses may be assessed to optimize therapeutic efficacy.

Certainly, to transition from bench to bedside, several challenges must still be addressed, including the inherent instability of RA, the need for scalable manufacturing processes, and further translational studies. Nonetheless, regarding the potential clinical applications of the long-circulating LAmB-RA formulation, our findings suggests that it may replace AmBisome in the treatment of CL and disseminated forms of leishmaniasis, such as DCL, MCL, and VL/HIV.

## 5 Conclusions

This work presents the successful development of a novel PEGylated liposomal formulation coencapsulating AmB and RA. The formulation showed suitable physicochemical properties and high drug encapsulation efficiencies.

The liposomal encapsulation significantly reduced AmB-induced hemolysis *in vitro* compared to Anforicin B, the micellar system. For the first time, the pharmacokinetics of AmB in LAmB and LAmB-RA were compared to AmBisome. PEGylated formulations exhibited higher *C*_max_, longer residence time, and reduced liver uptake, supporting enhanced delivery to lesion site favored by inflammation-enhanced vascular permeability in infected tissue.

In murine models, LAmB-RA significantly inhibited lesion progression in both models, with a more pronounced effect observed in the *L. major* model, whereas AmBisome only delayed lesion growth. In *L. amazonensis*-infected mice treated with LAmB-RA, the improved outcome was associated with a Th1-skewed immune response, as indicated by increased IFN-γ levels in spleen cell cultures. All treatments were well tolerated, with only mild renal toxicity observed.

The promising results obtained in the scope of this study support LAmB-RA as a potential strategy against leishmaniasis, a neglected disease affecting over 90 countries worldwide.

## Supplementary Material



## Data Availability

Data related to this paper may be found at 10.6084/m9.figshare.29835230.

## Notes

Additional materials and methods for SAXS analysis, pharmacokinetic study, and *in vivo* evaluation of liposomes’ therapeutic efficacy and toxicity; size distribution profiles, UV–Vis and circular dichroism analyses, the impact of different treatments on mice body weight, cytokine and chemokine profiles in liver lysates, and the macroscopic aspects of lesions in infected mice (PDF)
