# Supplementary material for: Long-Circulating Liposomes Codelivering Amphotericin B and Retinoic Acid for Cutaneous Leishmaniasis Treatment
Source: ACS Omega. 2025 Oct 7;10(41):48514–30. doi: 10.1021/acsomega.5c06156 (PMC12547526; doi:10.1021/acsomega.5c06156)
Supplement: Supplementary file 1 [file ao5c06156_si_001.pdf]

Supporting Information

Long-circulating liposomes co-delivering amphotericin B and retinoic acid for cutaneous leishmaniasis treatment

Thais T. Santos [a,b], Eduardo B. Lages [c], Tiago N. Q. Ricotta [c], Leandro G. de Oliveira [c], Guilherme S. Ramos [a], Julie Burlot [d], Sonia Abreu [e], Pierre Chaminade [e], François-Xavier Legrand [f], Pauline Tran [g], Claudine Deloménie [g], Virgínia M. R. Vallejos [a], Doumet Georges Helou [h], Raquel M. de Almeida [a], Celso M. Queiroz-Junior [a], Gabriel B. M. Teobaldo [i], Cristiano L. P. de Oliveira [i], Lucas A. M. Ferreira [c], Marta M. G. Aguiar [c*], Sébastien Pomel [b*], Frédéric Frézard [a*]

[a] Institute of Biological Sciences, Federal University of Minas Gerais (UFMG), 31270-901 Belo Horizonte, Brazil
[b] Université Paris-Saclay, CNRS BioCIS, 91400 Orsay, France
[c] Faculty of Pharmacy, Federal University of Minas Gerais (UFMG), 31270-901 Belo Horizonte, Brazil
[d] Université Paris-Saclay, UMS-IPSIT Animex, INSERM, CNRS, Ingénierie et Plateformes au Service de l'Innovation Thérapeutique, 91400 Orsay, France
[e] Lipides: Systèmes Analytiques et Biologiques, Université Paris-Saclay, 91400 Orsay, France
[f] Université Paris-Saclay, CNRS, Institut Galien Paris-Saclay, 91400 Orsay, France
[g] Université Paris-Saclay, UMS-IPSIT ACTAGen, INSERM, CNRS, Ingénierie et Plateformes au Service de l'Innovation Thérapeutique, 91400 Orsay, France
[h] Université Paris Cité, INSERM UMR1149, Centre de Recherche sur l'Inflammation (CRI), Paris 75018, France
[i] Instituto de Física, Universidade de São Paulo, 05508-0900 São Paulo, Brazil

***Corresponding authors:**
martagontijoa@gmail.com;
sebastien.pomel@universite-paris-saclay.fr;
frezard@icb.ufmg.br

Table of Contents

Additional Materials and Methods 3
1.1. Liposomes physicochemical characterization.. 3
1.1.1. Small-Angle X-ray Scattering (SAXS) Analysis.. 3
1.2. Pharmacokinetic study 3
1.2.1. Plasma sample preparation 3
1.2.2. Liver sample preparation.. 3
1.2.3. Bioanalytical method validation.. 3
1.3. *In vivo* assessment of the therapeutic efficacy and immunomodulation of liposomal formulations.. 4
1.3.1. Treatment efficacy.. 4
1.3.2. Cytokine profile determination... 5
1.3.3. Toxicity evaluation.. 5
References... 10

List of Tables

Table S1. MS/MS parameters used to quantify AmB in plasma and liver. 4

List of figures

Supplementary Figures.. 6
Figure S1. Representative intensity size distribution of PEGylated liposomal formulations... 6
Figure S2. AmB aggregation state by UV-Vis spectrophotometry and circular dichroism (CD) in different formulations... 7
Figure S3. Impact of treatment with different liposomal AmB formulations by IP route on body weight and macroscopic aspect of lesions in *L. major*-infected mice............................ 7
Figure S4. Impact of treatment with different liposomal AmB formulations by IP route on the cytokine and chemokine profile in liver lysates of *L. major*-infected mice... 8
Figure S5. Impact of treatment with different liposomal AmB formulations by IP route on body weight and macroscopic aspect of lesions in *L. amazonensis*-infected mice. 9

Additional Materials and Methods

1.1. Liposomes physicochemical characterization

1.1.1. Small-Angle X-ray Scattering (SAXS) Analysis

In this modeling approach [1] the bilayer electron density profile (EDP), which provides indication about the variation in the electron densities perpendicular to the bilayer surfaces, is described by a set of Gaussian functions. From the EDP it is possible to obtain the relative electron density contrast within the bilayer, overall bilayer thickness and indications about the bilayer asymmetry. The theoretical scattering intensity is given by the equation S1:

$$I(q) = \frac{1}{q^2} I_{EDP}(q) + back \qquad \text{(Eq. S1)}$$

Where IEDP(q) is the scattering intensity obtained from the electron density profile of the lipid bilayer. By a 'model free' modeling, the EDP that provides the best fit of the experimental data is obtained, without any specific hypothesis about the head or tail groups in the system composition. Mathematical details can be obtained in the original reference [1].

1.2. Pharmacokinetic study

1.2.1. Plasma sample preparation

Plasma sample preparation was carried out by protein precipitation according to the protocol proposed by Pippa et al. [2]. Plasma aliquots of 25 µL were prepared by adding 25 µL of Internal Standard (IS) in methanol (piroxicam, 140 ng/mL), 25 µL of methanol, and 100 µL of 0.1% formic acid in methanol. Tubes were shaken for 5 sec, then centrifuged at 4 °C for 15 min at 19,090 g. Next, 100 µL of the supernatant was transferred to the injection vial and mixed with 100 µL of 0.1% formic acid in water. 5 µL of the final mixture was subjected to chromatographic analysis. Standard calibration curves were prepared using plasma from untreated mice.

1.2.2. Liver sample preparation

Liver sample preparation was carried out using protein precipitation as described in the literature [3,4]. The liver was manually macerated using a glass rod to obtain a homogenate. In a first step, a liver aliquot (200 ± 10 mg) was collected, then 50 µL of IS (piroxicam, 1,120 ng/mL) and 350 µL of methanol were added. This mixture was vortexed for 10 s and centrifuged at 4 °C for 15 min at 19,090 g. After that, 200 µL of the supernatant was transferred to a tube, and 25 µL was used for the second step. The second step was similar to the plasma preparation described above.

1.2.3. Bioanalytical method validation

A bioanalytical method was validated to determine AmB concentration in plasma and liver, employing high-performance liquid chromatography Vanquish Flex (Thermo Scientific, Boston USA) coupled to a mass spectrometer (LC-MS/MS). The analyses were conducted using a C8 column (150 × 2.1 mm; 4 µm), mobile phase composed of Methanol: Acetonitrile: Water: Formic acid (15: 40: 45: 0.1), and a 0.5 mL/min flow rate. The mass spectrophotometer Orbitrap Exploris 120 (Thermo Scientific, Boston, USA) operating in heated electrospray ion source (HESI-II) mode was used. The quantification mode was targeted-tandem mass

spectrometry (t-MS2). Each targeted compound is detected in its chromatographic segment using an inclusion mass list. The quadrupole selects the precursor ions with an isolation window of $m/z = 2$.

MS/MS analysis was performed using the positive ionization mode. The capillary voltage for electrospray ionization was set at 3.80 kV. The ion transfer tube and vaporizer temperatures were kept constant at 350 °C and 345 °C, respectively. The sheath and auxiliary gas flow rates were set at 45 and 15, respectively. Normalized High Collision Energy Dissociation (HCD) was used for collision purposes, 11% for AmB and 39% for IS.

The mass resolution was set at 120,000. MS^2 spectrum was registered between m/z 200 and 955 for AmB and between m/z 50 and 363 for the IS. The ion m/z 906.48, corresponding to the $[M + H - H_2O]^+$ adduct of AmB, was selected as the precursor ion for targeted MS^2 analysis. This ion is commonly formed during electrospray ionization due to the spontaneous dehydration of the protonated molecule, a well-known behavior of AmB [5] . Due to its stability and specificity, the resulting fragment ion at m/z 743.40 was selected for quantification. The limit of quantification (LOQ) for AmB was established at 5 ng/mL. The signal of the daughter ion was extracted from the MS^2 spectrum (Table S1).

Table-S1. MS/MS parameters used to quantify AmB in plasma and liver.

Analyte	Parent Ion	Extracted ion mass range	HCD (%)
AmB	906.4846	743.370 – 743.430	11
IS	332.0700	95.058 – 95.062	39

1.3. In vivo assessment of the therapeutic efficacy and immunomodulation of liposomal formulations

1.3.1. Treatment efficacy

In CL murine model using *L. major,* mice were euthanized three days after the end of treatment, and skin samples around the lesions were collected to evaluate the parasitic load by quantifying the *Leishmania* genomes using real-time PCR (qPCR), as previously described [6]. DNA was extracted from mouse skin using the QIAamp DNA Mini Kit (Qiagen, Hilden, Germany), according to the manufacturer's instructions. Briefly, DNA extraction was done from skin samples (15 ± 5 mg) previously cut into tiny pieces, mixed with lysis buffer and Proteinase K, and incubated at 56 °C overnight. The concentration and purity of extracts were measured by UV spectrophotometry (absorbance at 280 and 260 nm) and DNA concentrations were adjusted to 5 ng/µL. For parasite load quantification, qPCR was performed in a CFX96 thermal cycler (Bio-Rad, USA) using the SSoADV Univer SYBR Green Supermix (Bio-Rad, USA), from 4 µL of each sample in a 10-µL reaction volume. Each oligonucleotide, i.e. forward (5'-AAGTGCTTTCCCATCGCAACT-3') and reverse (5'-GACGCACTAAACCCCTCCAA-3') primers, designed for amplifying a short fragment of 18S rDNA gene in *Leishmania* [6], was used at 0.5-µM final concentration. The standard curve was obtained from serial dilutions of known copy numbers of *Leishmania* genomes, using DNA extracted from a culture of *L. major* (MHOM/PT/92/CRE26). Data are presented as numbers of *Leishmania* genomes per µg of total DNA.

Parasite load was similarly assessed for the *L. amazonensis* murine model. Mice were euthanized three days after the end of treatment. The spleen was immediately removed to evaluate the parasitic load as described previously [7]. DNA was extracted using the PureLink Genomic DNA Mini Kit (Invitrogen, USA) according to the manufacturer's instructions. Briefly, DNA was extracted from an aliquot of spleen which was macerated before mixing with lysis buffer and Proteinase K, and incubated at 56 ºC overnight. The DNA concentrations were

measured by spectrophotometry (absorbance at 280 and 260 nm) and adjusted to 5 ng/µL. The qPCR was performed in a Quantstudio 3 real-time PCR system (Applied Biosystems) using the PowerUp SYBR Green Master Mix (Invitrogen, USA), from 1 µL of each sample in a 10-µL reaction volume containing 10 pmol of each oligonucleotide, i.e. forward (5'-CGT GGGGGAGGGGCGTTCT-3') and reverse (5'-CCGAAGCAGCCGCCCCTATT-3') primers constructed for amplification of the mini-circle region present in the kinetoplast DNA (kDNA) [7]. The standard curve was obtained with serial dilutions of known DNA concentrations of *L. amazonensis* (IFLA/BR/1967/PH8) extracted from promastigote culture. Data are presented as nanogram (ng) amount of *Leishmania* DNA per µg of total DNA.

1.3.2. Cytokine profile determination

For SLA preparation, *L. amazonensis* parasites obtained from cultures at the beginning of the stationary growth phase were washed twice with PBS by centrifugation at 1,540 g, at 4 °C, for 10 min. They were then subjected to seven freeze cycles in liquid nitrogen and thawing at 37 °C. The efficiency of parasite lysis was monitored by optical microscopy [8]. Protein dosage of the extracts obtained was performed using the Bradford method [9], using a commercial reagent (Sigma Aldrich, USA), according to the manufacturer's instructions. Cytokine assessment in the liver and spleen has been used in the literature to check the immunomodulatory effect of treatments and vaccines [10,11].

1.3.3. Toxicity evaluation

Animal blood was collected and centrifugated at 1,700 g for 10 min, and the plasma supernatant was collected for biochemical analyses. These analyses were performed in the Bioplus BIO-2000 semiautomatic analyzer (São Paulo, Brazil) using commercial kits and following the manufacturer's instructions. Kidneys were also collected for histopathological examination. Phosphate buffered formalin (10%) was used to fix the samples, followed by dehydration in alcohol and embedding in paraffin blocks. After obtaining organ sections, they were stained with hematoxylin and eosin (H&E) [12]. Images were made using a camera connected to an optical microscope (Olympus BX-40; Olympus, Tokyo, Japan).

Supplementary Figures

Results

			Size (d.n...	% Intensity:	St Dev (d.n...
**Z-Average (d.nm):**	124,0	**Peak 1:**	138,7	100,0	47,34
**PdI:**	0,092	**Peak 2:**	0,000	0,0	0,000
**Intercept:**	0,937	**Peak 3:**	0,000	0,0	0,000
**Result quality**	Good				

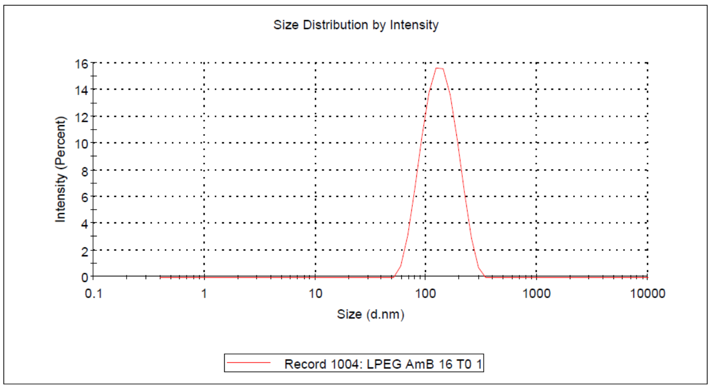

(A)

Results

			Size (d.n...	% Intensity:	St Dev (d.n...
**Z-Average (d.nm):**	118,7	**Peak 1:**	135,2	100,0	47,48
**PdI:**	0,114	**Peak 2:**	0,000	0,0	0,000
**Intercept:**	0,936	**Peak 3:**	0,000	0,0	0,000
**Result quality**	Good				

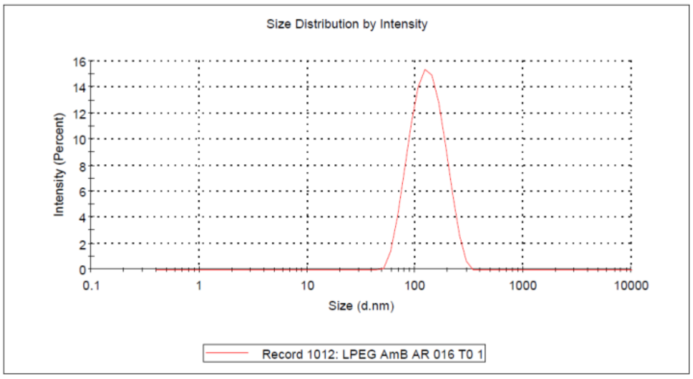

(B)

Figure S1. Representative intensity size distribution of PEGylated liposomal formulations. (A) LAmB and (B) LAmB-RA. Data obtained by dynamic light scattering at 25 °C and a fixed angle of 90° using a Zetasizer Nano ZS90. Samples were previously diluted 1:100 in deionized water.

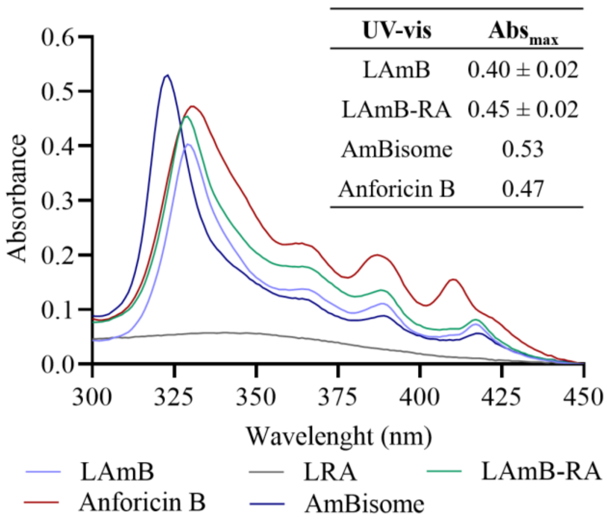

(A)

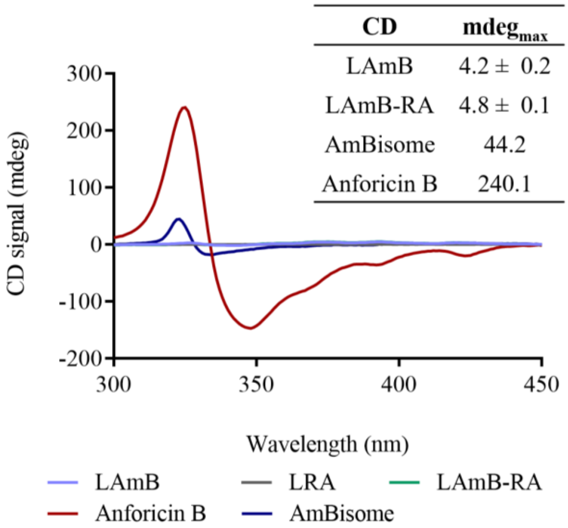

(B)

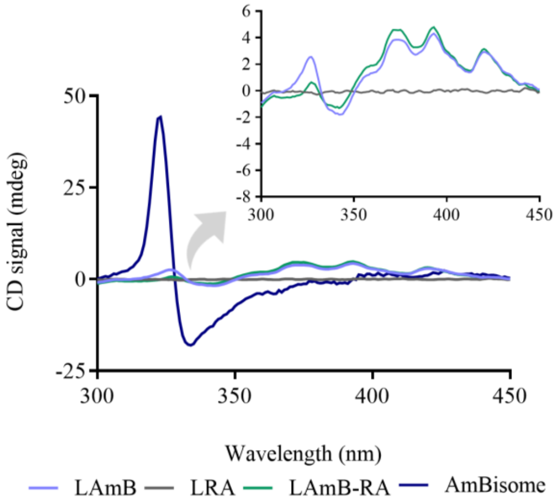

(C)

Figure S2. AmB aggregation state by UV-Vis spectrophotometry and circular dichroism (CD) in different formulations. (A) absorption spectra containing maximum absorption values; (B) CD spectra registered containing maximum mdeg values; (C) CD spectra without Anforicin B with inset showing a magnified view in the spectrum without AmBisome. The spectra were registered after 580-fold dilution in PBS (AmB 5 µg/mL). Maximum absorption wavelengths for UV-Vis and CD were: LAmB and LAmB-RA (329 and 393 nm); Anforicin B (330 and 325 nm); and AmBisome (323 nm for both methods).

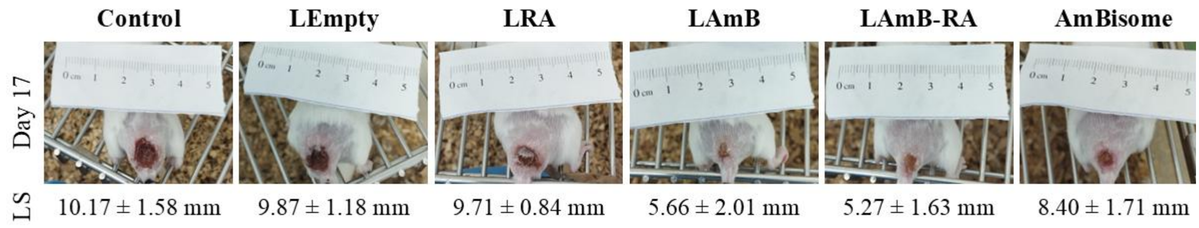

(A)

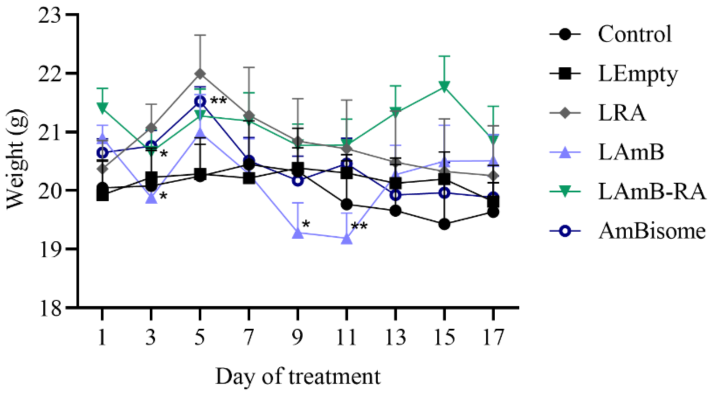

(B)

Figure S3. Impact of treatment with different liposomal AmB formulations by IP route on body weight and macroscopic aspect of lesions in *L. major*-infected mice. (A). LS is the lesion size mean ± SD on day 17 in each group. (B) Body weight of *L. major*-infected mice as a function of time. Data are mean + SEM (n = 8 for each group, and n = 10 for the Control) and analyzed through repeated measures two-way ANOVA followed by Dunnett's multiple comparison post-test. Significant differences were observed compared to Day 1 ($^{*}p < 0.05$, $^{**}p < 0.01$).

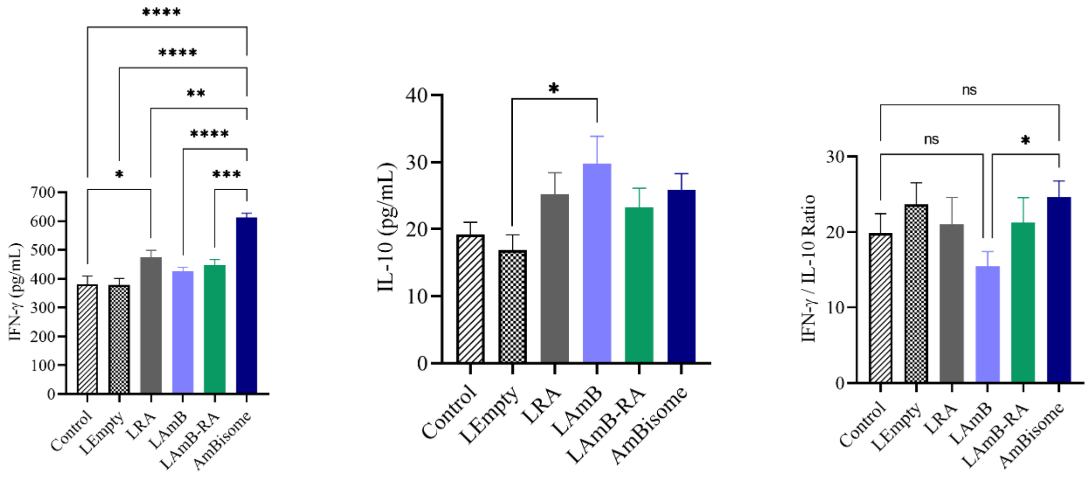

(A) (B) (C)

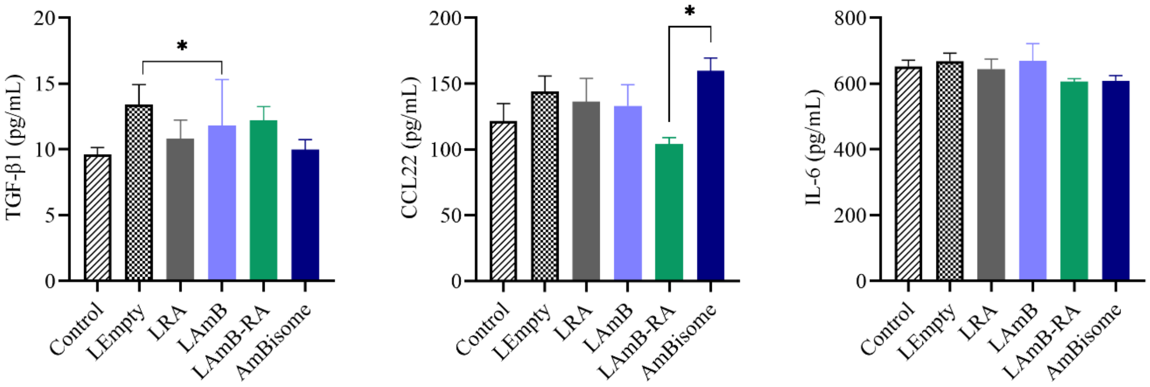

(D) (E) (F)

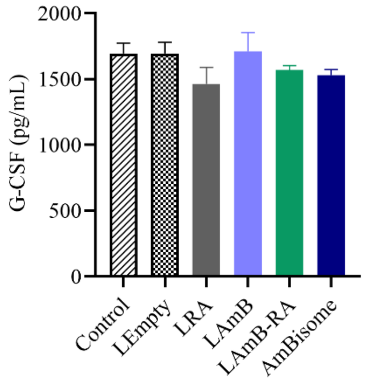

(G)

Figure S4. Impact of treatment with different liposomal AmB formulations by IP route on the cytokine and chemokine profile in liver lysates of *L. major*-infected mice. (A) IFN -γ, (B) IL-10, (C) IFN -γ / IL-10 Ratio, (D) TGF – β1 (E) CCL22, (F) IL-6, and (G) G-CSF. Data are mean ± SEM (n = 6) and analyzed through one-way ANOVA followed by Tukey's multiple comparison post-test. IFN-γ/L-10 ratio was analyzed through one-way ANOVA followed by Holm-Sidak's multiple comparison post-test. Significant differences were observed ($^{*}p < 0.05$, $^{**}p < 0.01$, $^{***}p < 0.001$, $^{****}p < 0.0001$).

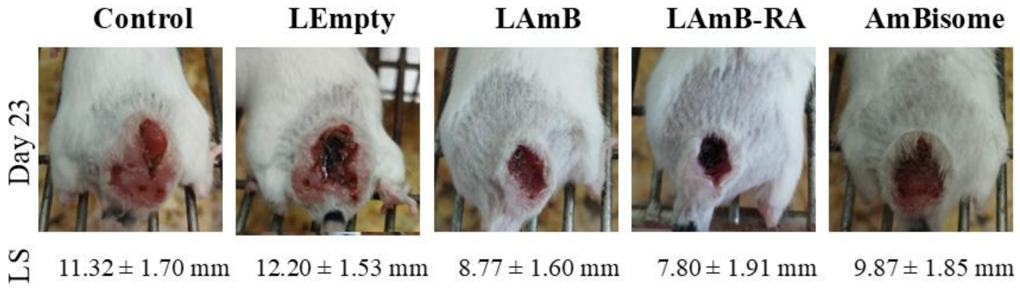

(A)

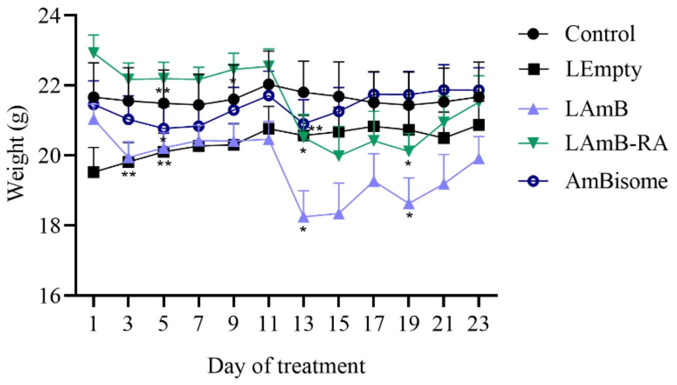

(B)

Figure S5. Impact of treatment with different liposomal AmB formulations by IP route on body weight and macroscopic aspect of lesions in *L. amazonensis*-infected mice. (A) LS is the lesion size mean ± SD on day 23 in each group. (B) Body weight of *L. amazonensis*-infected mice as a function of time. Data are mean + SEM (n = 7) and analyzed through repeated measures two-way ANOVA followed by Dunnett's multiple comparison post-test. Significant differences were observed compared to Day 1 ($^{*}p < 0.05$, $^{**}p < 0.01$).

References

(1) Oliveira, C. L. P.; Gerbelli, B. B.; Silva, E. R. T.; Nallet, F.; Navailles, L.; Oliveira, E. A.; Pedersen, J. S. Gaussian Deconvolution: A Useful Method for a Form-Free Modeling of Scattering Data from Mono- and Multilayered Planar Systems. *Applied Crystallography* **2012**, *45* (6), 1278–1286.

(2) Pippa, L. F.; Marques, M. P.; Silva, A. C. T. da; Vilar, F. C.; de Haes, T. M.; Fonseca, B. A. L. da; Martinez, R.; Coelho, E. B.; Wichert-Ana, L.; Lanchote, V. L. Sensitive LC-MS/MS Methods for Amphotericin B Analysis in Cerebrospinal Fluid, Plasma, Plasma Ultrafiltrate, and Urine: Application to Clinical Pharmacokinetics. *Front Chem* **2021**, *9* (November), 1–14. https://doi.org/10.3389/fchem.2021.782131.

(3) Vogelsinger, H.; Weiler, S.; Djanani, A.; Kountchev, J.; Bellmann-Weiler, R.; Wiedermann, C. J.; Bellmann, R. Amphotericin B Tissue Distribution in Autopsy Material after Treatment with Liposomal Amphotericin B and Amphotericin B Colloidal Dispersion. *Journal of Antimicrobial Chemotherapy* **2006**, *57* (6), 1153–1160. https://doi.org/10.1093/jac/dkl141.

(4) Wang, D.; Zhang, W.; Ju, J. X.; Wang, L. J.; Huang, R. Y.; Xu, Y. F.; Zhang, H. L.; Qi, J. L. Gender Differences in Acute Toxicity, Toxicokinetic and Tissue Distribution of Amphotericin B Liposomes in Rats. *Toxicol Lett* **2021**, *338* (November 2020), 78–84. https://doi.org/10.1016/j.toxlet.2020.12.004.

(5) Dar, M. J.; Khalid, S.; McElroy, C. A.; Satoskar, A. R.; Khan, G. M. Topical Treatment of Cutaneous Leishmaniasis with Novel Amphotericin B-Miltefosine Co-Incorporated Second Generation Ultra-Deformable Liposomes. *International Journal of Pharmaceutics*. 2020. https://doi.org/10.1016/j.ijpharm.2019.118900.

(6) Malli, S.; Pomel, S.; Ayadi, Y.; Deloménie, C.; Da Costa, A.; Loiseau, P. M.; Bouchemal, K. Topically Applied Chitosan-Coated Poly(Isobutylcyanoacrylate) Nanoparticles Are Active Against Cutaneous Leishmaniasis by Accelerating Lesion Healing and Reducing the Parasitic Load. *ACS Appl Bio Mater* **2019**, *2* (6), 2573–2586. https://doi.org/10.1021/acsabm.9b00263.

(7) Carregal, V. M.; Lanza, J. S.; Souza, D. M.; Islam, A.; Demicheli, C.; Fujiwara, R. T.; Rivas, L.; Frézard, F. Combination Oral Therapy against Leishmania Amazonensis Infection in BALB/c Mice Using Nanoassemblies Made from Amphiphilic Antimony(V) Complex Incorporating Miltefosine. *Parasitol Res* **2019**, *118* (10), 3077–3084. https://doi.org/10.1007/s00436-019-06419-2.

(8) Afonso, L. C. C.; Scott, P. Immune Responses Associated with Susceptibility of C57BL/10 Mice to Leishmania Amazonensis. *Infect Immun* **1993**, *61* (7), 2952–2959. https://doi.org/10.1128/iai.61.7.2952-2959.1993.

(9) Bradford, M. M. A Rapid and Sensitive Method for the Quantitation of Microgram Quantities of Protein Utilizing the Principle of Protein-Dye Binding. *Anal Biochem* **1976**, *72* (1–2), 248–254.

(10) Hernandez, F. M. de O.; Santos, M. O.; Venturin, G. L.; Bragato, J. P.; Rebech, G. T.; Melo, L. M.; Costa, S. F.; de Freitas, J. H.; Siqueira, C. E.; Morais, D. A.; others. Vitamins A and D and Zinc Affect the Leshmanicidal Activity of Canine Spleen Leukocytes. *Animals* **2021**, *11* (9), 2556.

(11) Helou, D. G.; Mauras, A.; Fasquelle, F.; Lanza, J. S.; Loiseau, P. M.; Betbeder, D.; Cojean, S. Intranasal Vaccine from Whole Leishmania Donovani Antigens Provides Protection and Induces Specific Immune Response against Visceral Leishmaniasis. *PLoS Negl Trop Dis* **2021**, *15* (8), 1–19. https://doi.org/10.1371/journal.pntd.0009627.

(12) Coelho, L. D.; Souza, M. M. D.; Cassali, G. D.; Silva, R. A.; Paiva, M. J. N.; Barros, A. L. B.; Teixeira, E. M.; Silveira, J. N.; Coelho, P. M. Z.; Aguiar, M. M. G.; Oliveira, M. C. Emetic Tartar-Loaded Liposomes as a New Strategy for Leishmaniasis Treatment. *Pharmaceutics* **2023**, *15* (3), 1–14. https://doi.org/10.3390/pharmaceutics15030904.
